# Characterization and Comparative Genomic Analysis of Three Virulent *E. coli* Bacteriophages with the Potential to Reduce Antibiotic-Resistant Bacteria in the Environment

**DOI:** 10.3390/ijms24065696

**Published:** 2023-03-16

**Authors:** Paulina Śliwka, Beata Weber-Dąbrowska, Maciej Żaczek, Marta Kuźmińska-Bajor, Izabela Dusza, Aneta Skaradzińska

**Affiliations:** 1 Department of Biotechnology and Food Microbiology, Wrocław University of Environmental and Life Sciences, 50-375 Wrocław, Poland; 2 Bacteriophage Laboratory, Ludwik Hirszfeld Institute of Immunology and Experimental Therapy, Polish Academy of Sciences, 53-114 Wrocław, Poland; 3 Phage Therapy Unit, Ludwik Hirszfeld Institute of Immunology and Experimental Therapy, Polish Academy of Sciences, 53-114 Wrocław, Poland

**Keywords:** bacteriophages, antibiotic resistance, ESBL/AmpC *E. coli*, lytic activity, genome sequencing, biocontrol, jumbo phage

## Abstract

The emerging global crisis of antibiotic resistance demands new alternative antibacterial solutions. Although bacteriophages have been used to combat bacterial infections for over a century, a dramatic boost in phage studies has recently been observed. In the development of modern phage applications, a scientific rationale is strongly required and newly isolated phages need to be examined in detail. In this study, we present the full characterization of bacteriophages BF9, BF15, and BF17, with lytic activity against extended-spectrum β-lactamases (ESBLs)- and AmpC β-lactamases (AmpC)-producing *Escherichia coli*, the prevalence of which has increased significantly in livestock in recent decades, representing a great hazard to food safety and a public health risk. Comparative genomic and phylogenetic analysis indicated that BF9, BF15, and BF17 represent the genera *Dhillonvirus*, *Tequatrovirus,* and *Asteriusvirus*, respectively. All three phages significantly reduced in vitro growth of their bacterial host and retained the ability to lyse bacteria after preincubation at wide ranges of temperature (−20–40 °C) and pH (5–9). The results described herein indicate the lytic nature of BF9, BF15, and BF17, which, along with the absence of genes encoding toxins and bacterial virulence factors, represents an undoubted asset in terms of future phage application.

## 1. Introduction

Antimicrobial resistance in bacteria is a major and growing concern worldwide. Recent estimates show that more than 670,000 infections caused by multidrug-resistant bacteria are reported in Europe alone, of which more than 33,000 are fatal, generating healthcare costs of up to EUR 1.1 billion each year [1]. For decades, the inappropriate and uncontrolled use of antibiotics in the form of both preventive measures and veterinary medicine has contributed to the selection of resistant *Enterobacteriaceae* in animal husbandry [2]. Intensive farming promotes the rapid spread of bacteria among animals and favors the diffusion of resistance along the food production chain [3,4,5]. According to EARS-Net, more than half of the *E. coli* isolates were resistant to at least one antimicrobial group under surveillance, including resistance to β-lactam agents triggered by encoded enzymes called extended-spectrum β-lactamases (ESBLs) and AmpC β-lactamases (AmpC) [1]. The literature confirms a high burden of ESBL and/or AmpC-producing *E. coli* in food-producing animals, highlighting the need for an interdisciplinary environmental control system [6,7,8,9].

At the end of the “antibiotic era”, bacteriophages are considered one of the most promising strategies against resistant bacteria, and the effectiveness of phages against ESBL/AmpC-producing *E. coli* associated with animal production has been reported [10,11,12,13,14]. Bacteriophages might therefore play a vital role in preventing transmission of foodborne pathogens from food-producing animals to humans as part of the key actions of the One Health initiative targeting the environment, human, and animal health [15].

Moreover, in our previous research, we hypothesized that the role of phages may not be limited to directly target the pathogens, but bacterial viruses may also be applied against the spread of bacteria with antibiotic-resistance genes in animal production [16]. In that study, we isolated 17 *E. coli*-specific bacteriophages from samples taken on Polish pig farms. Phages were isolated using bacterial hosts with undefined antibiotic resistance profiles, and subsequently their activity was tested against 104 ESBL/AmpC *E. coli* strains isolated from pig farms and 51 ESBL/AmpC *E. coli* strains from turkey farms. The strains were isolated from the breeding farms in Germany and the presence of the β-lactamase genes blaCTX, blaTEM, blaSHV, and the CIT-type AmpCs (e.g., CMY-2) was confirmed by real-time PCR as described by Roschanski et al. [17]. The analysis of the lysis effect of the pool of the viruses revealed that the phages BF9, BF15, and BF17 might be applied simultaneously as a phage cocktail to efficiently control resistant *E. coli*. The proposed cocktail of BF9, BF15, and BF17 lysed 80.8% and 74.5% of the tested strains from pig and turkey farms, respectively [16]. In light of the above, here we characterized phages BF9, BF15, and BF17 through a detailed genomic annotation and based on the general aspects of their biological properties prior to desired downstream applications as novel biocontrol agents.

## 2. Results and Discussion

### 2.1. Morphology and Properties of Phage Virions and Plaques

**Figure 1:**
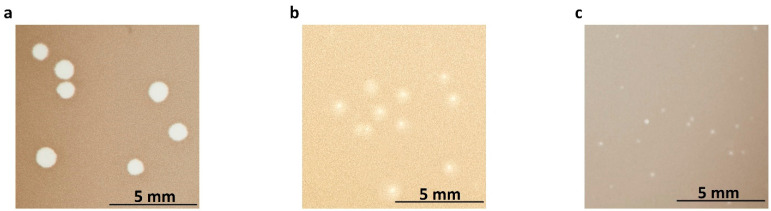

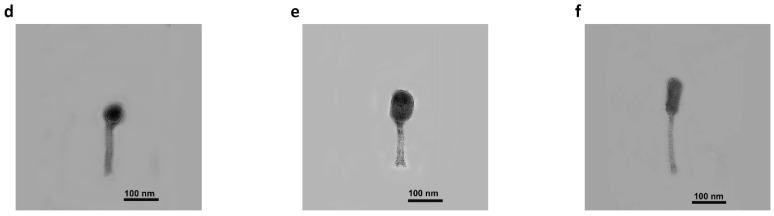
Virions and plaque morphology of phages BF9, BF15, and BF17. Upper panels (**a**–**c**) represent plaques of BF9 (**a**), BF15 (**b**), and BF17 (**c**) formed in double-layer agar plates with respective host strains. Lower panels (**d**–**f**) represent TEM micrographs of phages BF9 (**d**), BF15 (**e**), and BF17 (**f**).

After propagation on respective host strains, all bacteriophages formed homogeneous plaques varying in size and turbidity (Figure 1a–c). Bacteriophage BF9 formed larger and clear plaques with a diameter of 1.12 ± 0.17 mm (Figure 1a). Transmission electron microscopy (TEM) showed that bacteriophage BF9 has a long (approx. 83 nm) tail and a relatively small, isometric head measuring 34 × 31 nm (Figure 1d). Plaques with a diameter of 0.67 ± 0.26 mm surrounded by a halo zone were observed for bacteriophage BF15 (Figure 1b). BF15 has an elongated capsid with a diameter of approximately 36 nm and a length of about 57 nm, with a tail of about 78 nm (Figure 1e). Phage BF17 produced transparent, round plaques with a much smaller diameter of approximately 0.34 ± 0.16 mm (Figure 1c). Small plaques have been reported for large phages with a genome exceeding 200 kbp and described in the literature as jumbo phages [18]. The BF17 phage is distinguished by the largest capsid (62 × 23 nm) and a long tail (approx. 99 nm). TEM micrographs indicate that all phages belong to the class *Caudoviricetes*.

### 2.2. General Features of Genomes

Basic genomic characteristics indicate large variations between phages in both genome type and size, as well as predicted open reading frame (ORF) number. The general features of the bacteriophage genomes are presented in Table S1. All phage genomes are double-stranded linear molecules. PhageTerm analysis revealed that the packaging of genetic material in BF9 and BF15 virions follow a headful (pac) model of the permuted genome, similar to phage P1-type mechanism [19]. The genome of bacteriophage BF9 is 44,963 bp in length with a high G+C content of 54.5%, 58 predicted genes, and no tRNAs. The BF15 genome is composed of 167,801 bp (G+C content 35.4%) and 267 ORFs, of which 55% were assigned to genes encoding putative functional proteins and 8 putative tRNA genes with different specificities. BF17’s genome is 383,493 bp in length. The pattern of DNA packaging into the BF17 capsid predicts the presence of long direct terminal repeats (DTR) of ~20.5 kb (Figure S1), a characteristic also found in previously published jumbo phages [20,21]. A total of 656 ORFs and 6 tRNAs were determined in the genome of BF17, but no functional annotation were assigned to the DTR region [22]. Most ORFs were predicted as hypothetical proteins with unknown functions. Genes with predicted protein function were assigned to five functional modules: (I) DNA replication, transcription, translation, and nucleotide metabolism, (II) structure and packaging/morphogenesis, (III) host lysis, (IV) restriction-modification system, (V) additional functions. Detailed information about the identified ORFs is shown in Table S2. No genes coding for lysogeny, toxins, or other virulence factors were identified in the genomes of bacteriophages BF9, BF15, and BF17.

### 2.3. Comparative Genomics of Phages BF9, BF15, and BF17

**Figure 2:**
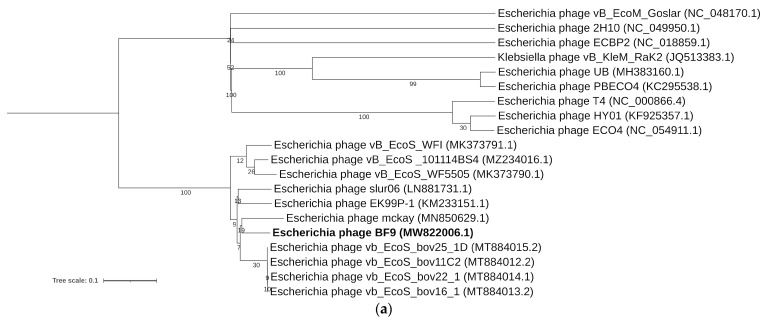

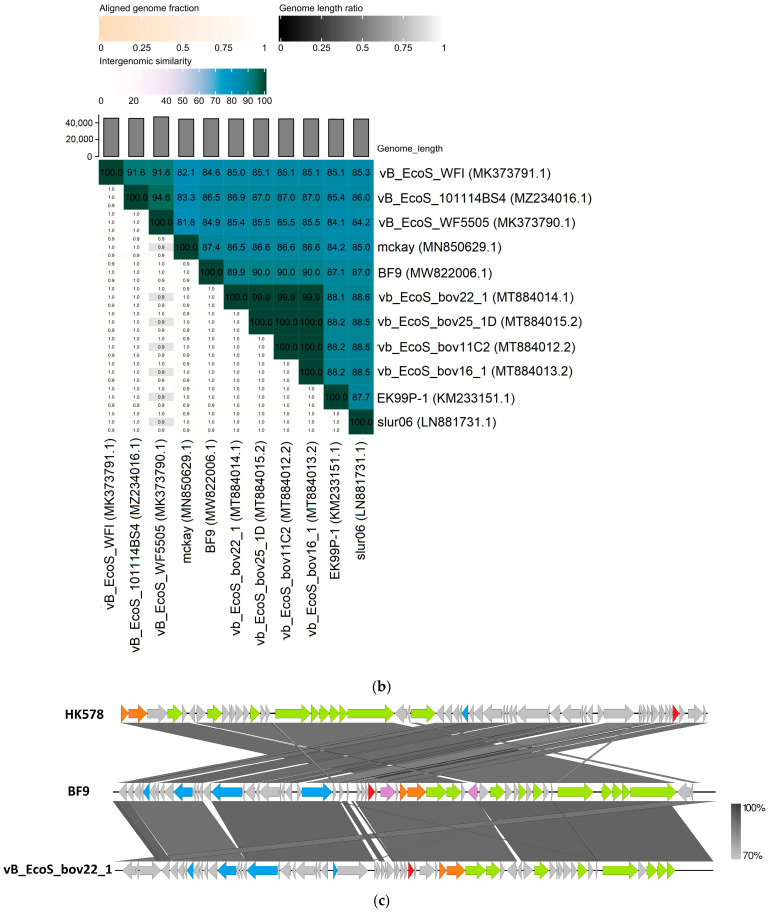
Genomic and phylogenetic analysis of bacteriophage BF9. (**a**) Phylogenetic tree of BF9 (in bold) generated by VICTOR using the whole-genome sequences of phage homologs in BLASTn. The numbers above branches are GBDP pseudo-bootstrap support values from 100 replications. (**b**) VIRIDIC heatmap based on intergenomic similarities between BF9 and closest homologs in BLASTn. The number in the chart represents the similarity percentage. (**c**) Genome comparison of BF9 with closely related members of the *Dhillonvirus* genus. The grey shading indicates sequence similarities between the genomes. The predicted functions of proteins are indicated by different colors: phage morphogenesis and virion structure (green arrows), DNA packaging (orange arrows), host lysis (red arrows), phage DNA replication and modification (blue arrows), and additional functions (purple arrows). Grey arrows represent ORFs of unknown functions.

Both the phylogenetic tree (Figure 2a) and intergenomic similarity heatmap (Figure 2b) showed that BF9 is a new member of the *Dhillonvirus* genus within *Caudoviricetes*, as proposed by ICTV [23]. Phages within this cluster exhibit the morphology of siphoviruses and can infect a range of *Enterobacteriaceae* hosts [24]. Other members of this taxon have a genome of comparable size (42–47 kb), about 54.5% G+C pair content, and no tRNA genes [25,26]. Overall, pairwise sequence identity between the phage BF9 and other related dhillonviruses was 84.6–90% (Figure 2b). The genome comparison showed high similarity among phages BF9 and vB_EcoS_bov22_1 (MT884014; 97% coverage and 93.01% identity), and a similar genome arrangement based on the putative function of predicted genes (Figure 2c). Lower overall similarities and greater divergence in genome topology were found between BF9 and phage HK578 (JQ086375; 84% coverage and 90.37% identity), a well-characterized type species of the *Dhillonvirus* genus. However, CoreGenes5.0 indicates that BF9 shares at least 46 homologs with HK578 (Table S3). Bacteriophages BF9, vB_EcoS_bov22_1, and HK578 feature high homology in sequences coding proteins thought to be involved in the virion structure and packaging. In our examples, we identify high nucleotide similarity in the genes of tail length tape measure protein (90.74% on average), head morphogenesis protein (93.3–95.5%), and both subunits of terminase (98% and 96% on average). The phage tail fiber protein encoded by ORF4 is highly similar (94.43% identity) to the gp22 of HK578, but the genome of BF9 lacks similarity in another sequence coding for tail fiber (gp25), which may have an impact on the divergent host range of this phages [25]. Furthermore, ORF28 encoding phage BF9 lysozyme was found in all genomes (gp57 in HK578 and gp56 in vB_EcoS_bov22_1) and displayed significant similarity (95.14–96.32%). In the comparison, regions of moderate similarity can also be observed, but they mostly correspond to the hypothetical proteins.

**Figure 3:**
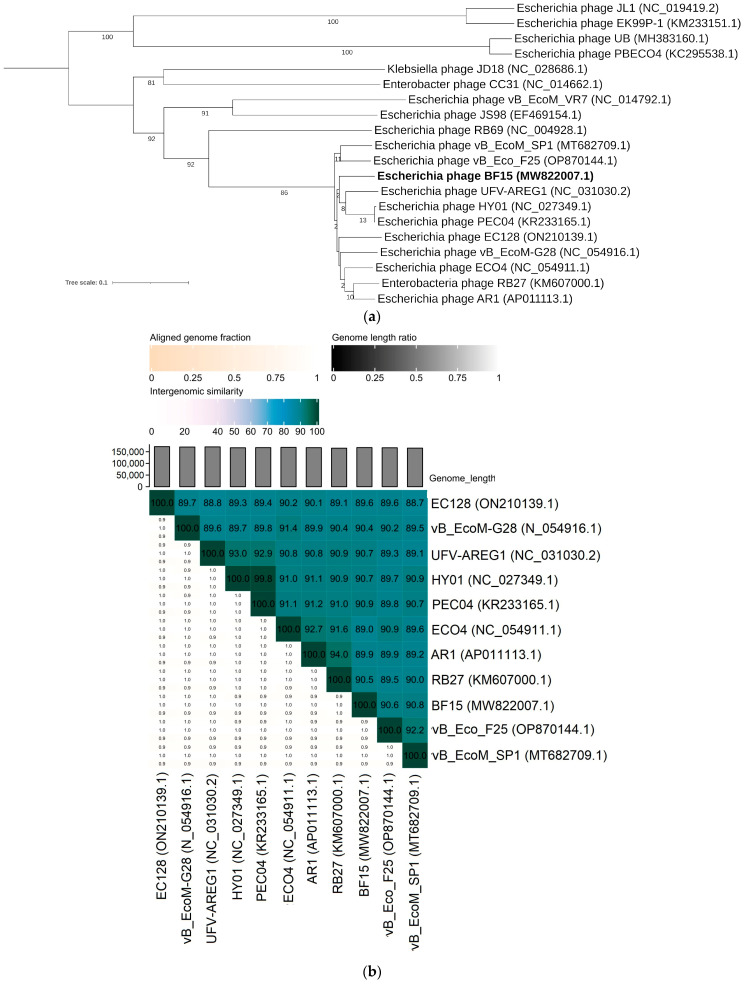

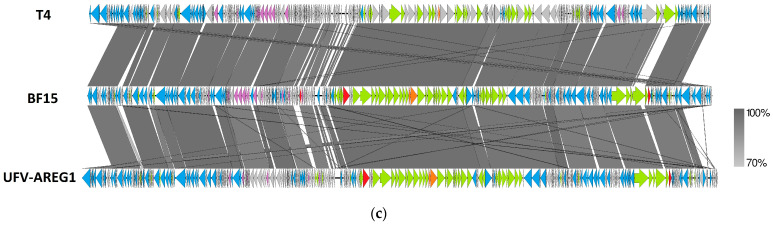
Genomic and phylogenetic analysis of bacteriophage BF15. (**a**) Phylogenetic tree of BF15 (in bold) generated by VICTOR using the whole-genome sequences of phage homologs in BLASTn. The numbers above branches are GBDP pseudo-bootstrap support values from 100 replications. (**b**) VIRIDIC heatmap based on intergenomic similarities between BF15 and closest homologs in BLASTn. The number in the chart represents the similarity percentage. (**c**) Genome comparison of BF15 with closely related members of the *Tequatrovirus* genus. The grey shading indicates sequence similarities between the genomes. The predicted functions of proteins are indicated by different colors: phage morphogenesis and virion structure (green arrows), DNA packaging (orange arrows), host lysis (red arrows), phage DNA replication and modification (blue arrows), and additional functions (purple arrows). Grey arrows represent ORFs of unknown functions.

In the whole-genome tree, BF15 formed a monophyletic group (92% bootstrap support) with members of the genus *Tequatrovirus* of the subfamily *Tevenvirinae*. The *Tequatrovirus* phages are among the most widely studied phages, isolated from many environments, confirmed to be lytic and safe for therapeutic use (Figure 3a) [27,28]. BF15 was found to be closest with phages UFV-AREG1 (KX009778.1), PEC04 (KR233165.1), and HY01 (KF925357.1), and high pairwise intergenomic similarity (<91%) seems to be consistent with VIRIDIC clustering (Figure 3b). Strong similarities to phage UFV-AREG1 at the nucleotide level (95% coverage, 91.8% identity) and in regard to its genomic organization were detected by genome comparison visualized using Easyfig (Figure 3c). The genome organization of both phages is reminiscent of the most well-known *Tevenvirinae,* phage T4 (NC_000866) (Figure 3c), where genes are clustered to form very similar modules of specific functions [29]. The analysis reveals that phages BF15 and T4 share 241 homologous genes in common (Table S3). Large numbers of conserved ORFs are located in the module of genes involved in virion structure and morphogenesis. No differences were found in the arrangement of genes encoding for major capsid proteins, tail proteins, or complex baseplate structure. However, we found a less homologous fragment (60% and 74.5% at nucleotide and amino acid level, respectively) between BF15 (ORF231) and T4 (gp34) with the same predicted function of the long tail fiber subunit responsible for host cell recognition. The conservation of genes among the host lysis module comprises putative lysozymes (90.1–97.28%) and holin (99.39%). BF15 has been found to possess high average identity in sequences encoding enzymes necessary for DNA synthesis shared with T4 and UFV-AREG1, such as DNA polymerase (97%), helicase (95.5%), DNA topoisomerase (97.2%), and DNA ligase (96.6%). Most of the differences in genome content between T4, BF15, and UFV-AREG1 were identified in genes that have not been functionally described to date.

**Figure 4:**
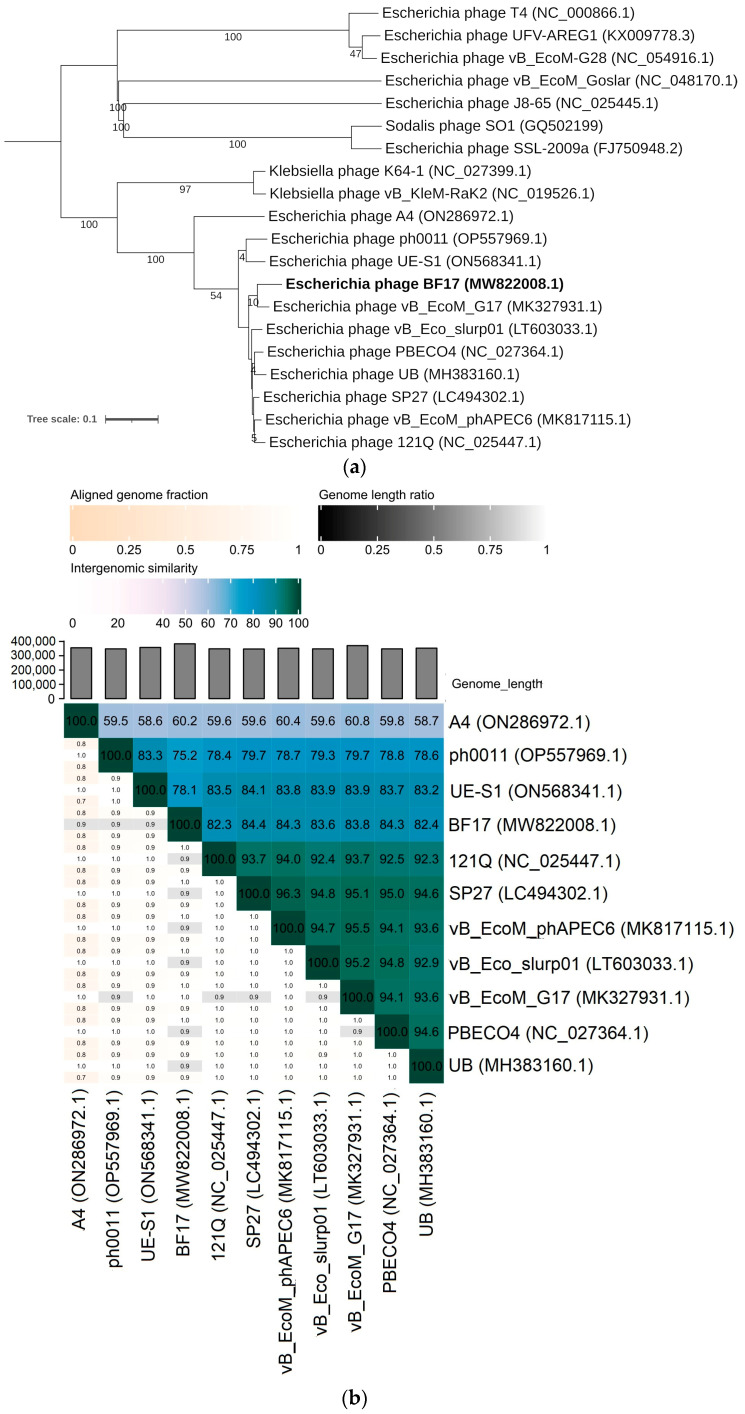

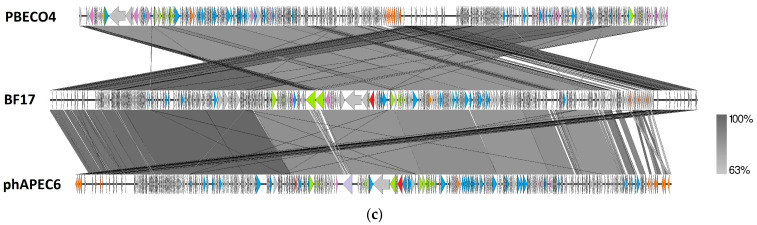
Genomic and phylogenetic analysis of bacteriophage BF17. (**a**) Phylogenetic tree of BF17 (in bold) generated by VICTOR using the whole-genome sequences of phage homologs in BLASTn. The numbers above branches are GBDP pseudo-bootstrap support values from 100 replications. (**b**) VIRIDIC heatmap based on intergenomic similarities between BF17 and closest homologs in BLASTn. The number in the chart represents the similarity percentage. (**c**) Genome comparison of BF17 with closely related members of the *Asteriusvirus* genus. The grey shading indicates sequence similarities between the genomes. The predicted functions of proteins are indicated by different colors: phage morphogenesis and virion structure (green arrows), DNA packaging (orange arrows), host lysis (red arrows), phage DNA replication and modification (blue arrows), and additional functions (purple arrows). Grey arrows represent ORFs of unknown functions.

The VICTOR analysis positioned BF17 in the single cluster of other *Escherichia*-specific phages where the jumbo phage AR (ON586972.1) is the outermost outlier (69% bootstrap value) (Figure 4a). Phages located on this branch are classified among the *Asteriusvirus* genus and have a genome of more than 200 kb [24]. Moreover, this clade showed significant divergence from the branch formed by several *Klebsiella*-specific jumbo phages of the *Alcyoneusvirus* genus. The latter also have the lowest degree of nucleotide homology with other compared genomes (30.2–62.7% intergenomic similarity) (Figure 4b). It is worth noting that jumbo phages have been described in divergent genera and do not constitute a uniform evolutionary group, which suggests the independent formation of large genomes in different phage groups [30]. According to ICTV guidelines, over 70% of the intergenomic similarity of BF17 with other representatives of *Asteriusvirus* may support the proposed classification [23]. In contrast to BF9 and BF15, BF17 shows an atypical genome arrangement (Figure 4c), a feature observed in other jumbo phages reported in the literature (reviewed in Yuan and Gao [18]). Their genome differs from the modular structure of genes that are expressed in a known time-dependent manner. Moreover, due to the genome size, jumbo phages are distinguished primarily by a large number of predicted ORFs, but the functional annotations have been attributed to a small fraction of the hypothetical genes [31,32,33,34]. The specific function of the vast majority of predicted proteins remains unknown, which constitutes the greatest limitation in accurately characterizing large phages. Only 10% of BF17 coding sequences have their homology counterparts in the NCBI GenBank database (Table S1). Based on CoreGene5.0, BF17 shares 569 homologous genes in common with phage PBECO4 (KC295538.1), mostly composed of genes of unknown function (Table S3).

Among the structural and morphogenesis genes cluster of the BF17 genome, it lacked the gene coding for the small terminase subunit. Instead, we identified two sequences coding for the large subunit of the terminase (ORF361 and ORF362). The ORF362 shared high nucleotide homology (85.33% on average) with PBECO4 and vB_EcoM_phAPEC6. Furthermore, in BF17 and compared genomes are noted copies of the chromosome condensation protein involved in the organization of large DNA molecules in the capsid. For example, ORF593 displays a high homology to PBECO4 (gp289) and vB_EcoM_phAPEC06 (gp549) of 92.76% and 95.53% sequence identity, respectively. Sequence conservation is also identified for the structural proteins with lysozyme activity involved in bacterial wall lysis (ORF271, ORF325, ORF326). The ORF326 is relatively similar to baseplate hub subunit and tail lysozyme of phage PBECO4 (gp534) (83.5% and 92.78% similarity at nucleotide and amino acid level, respectively). The longest sequences of BF17 coding tail fiber subunits (ORF310, ORF311) were partially conserved in phage phAPEC6 and PBECO4, but the remaining nucleotide sequence identity is low (32% on average) and no similarities were found at the amino acid level. Interestingly, high homology (95.8%) was observed between the genes located in BF17 (ORF313) and PBECO4 (gp547) likely involved in the degradation of the bacterial cell wall polysaccharide component [35]. Hydrolytic activity towards colanic acid has also been attributed to the IME-EC2 bacteriophage having the ability to degrade the bacterial envelope and biofilms [36]. Anti-biofilm potential of BF17 should be the subject of future investigations as the structure of biofilm may facilitate the transmission of ESBL genes and the persistence of antibiotic-resistant bacteria, especially in food processing environments [37].

### 2.4. Determination of Bacteriolytic Activity at Different Multiplicities of Infection

**Figure 5:**
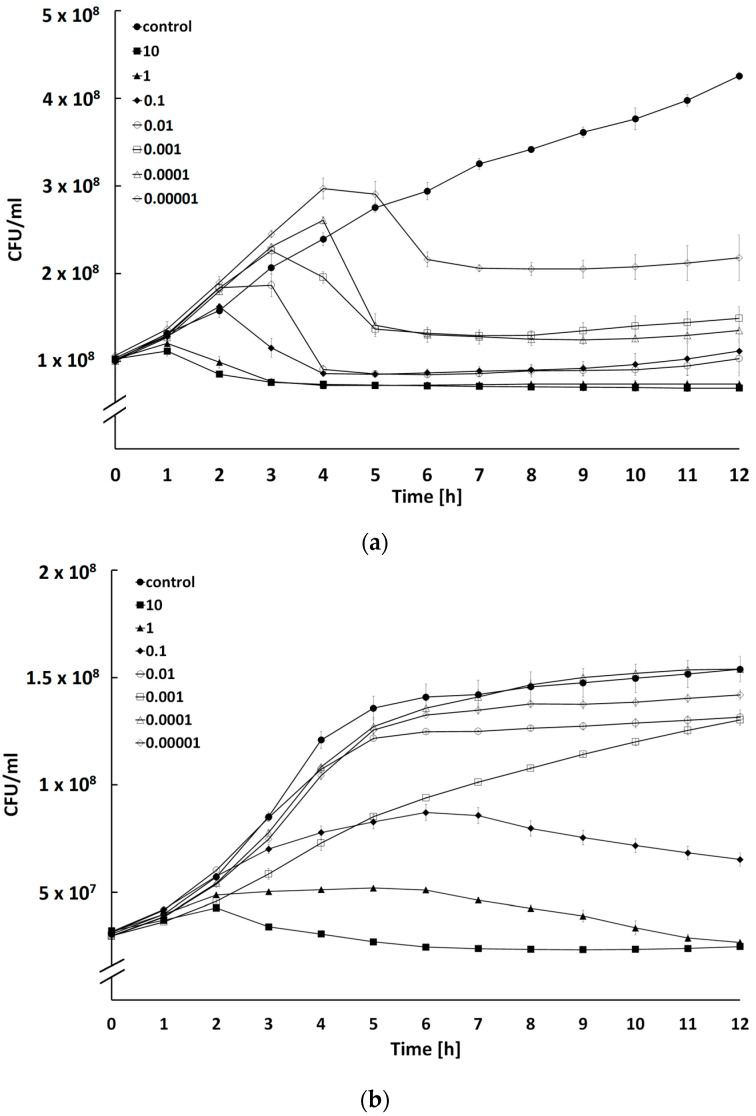

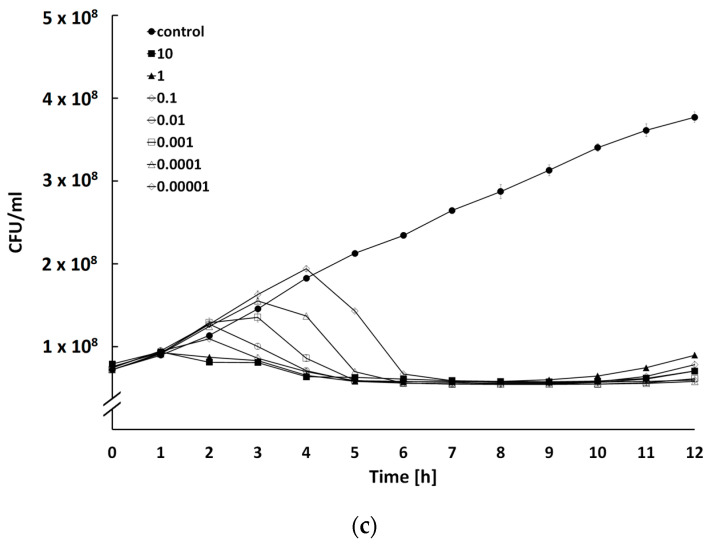
Effects of bacteriophages BF9 (**a**), BF15 (**b**), and BF17 (**c**) on count of the host strain. The strains were treated with phages at different MOIs. Each data point represents the mean and standard deviation of three different experiments.

The growth curves of the host strain after treatment with BF9, BF15, or BF17 showed differential bactericidal activity of phages with a series of MOIs (Figure 5). The dose-dependent relationship between MOI and bacterial counts was particularly evident for BF15 (Figure 5b). An increase in the phage concentration significantly limited the growth of a bacterial host. For phage BF9, the number of bacteria dropped after 6 h at all tested MOIs (Figure 5a). However, we observed less effective control of bacterial expansion with the use of lower MOIs (0.00001–0.1). These findings suggest the use of higher phage concentrations to compensate for the reduction of host growth. Another explanation could be the development of slow-growing phage-resistant mutants during infection [38]. Interestingly, the partial regrowth of BF17-treated bacteria occurred at much higher MOIs (>1) (Figure 5c). It has been suggested that the in vitro growth of bacteria insensitive to phages is driven by the selective pressure of high concentrations of lytic phages [39]. Thus, further increasing phage loads would not exert a beneficial impact on the antibacterial efficiency. An optimal MOI should be taken into consideration to maximize the infectivity of the phages.

### 2.5. Influence of Temperature and pH on Activity of Bacteriophages

**Figure 6:**
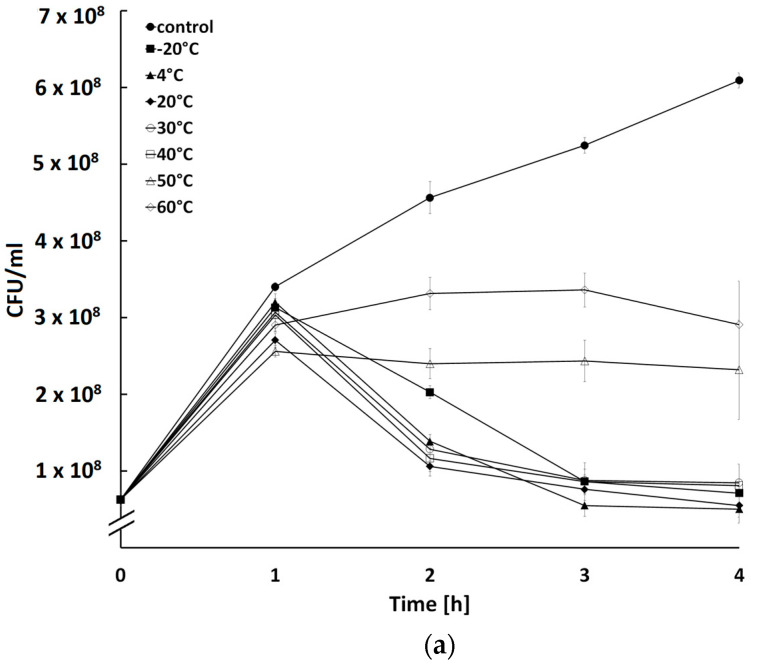

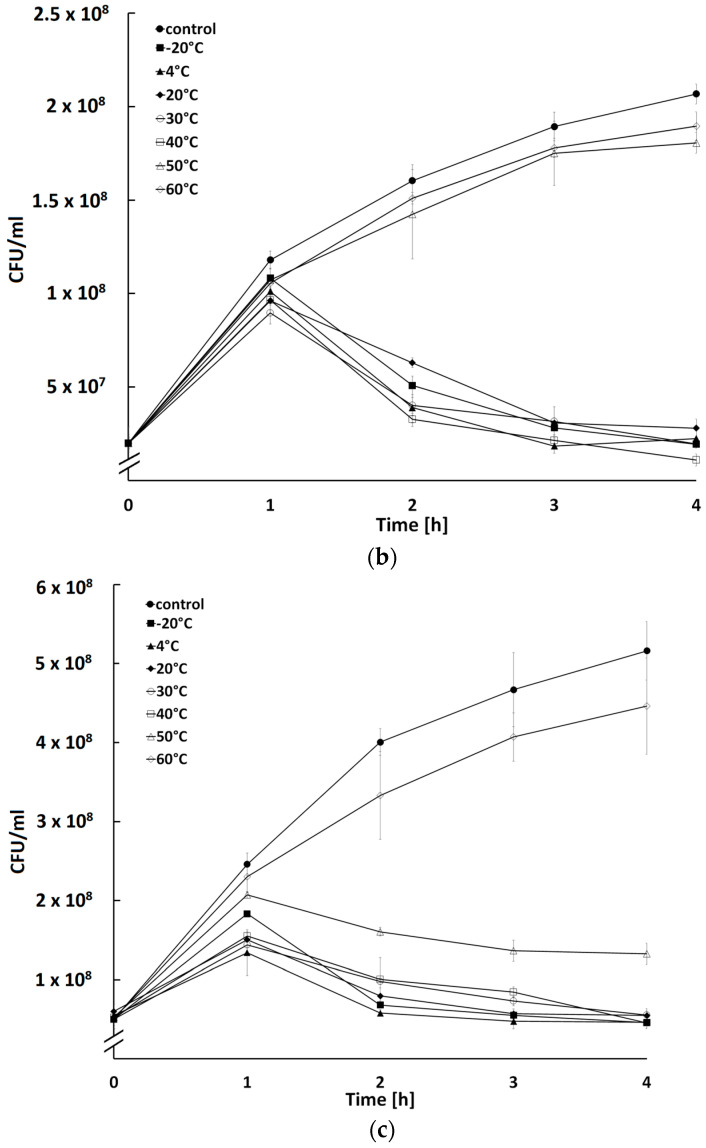
Bactericidal activity of phages BF9 (**a**), BF15 (**b**), and BF17 (**c**) pre-incubated at different temperatures on count of the host strain. Each data point represents the mean and standard deviation of three different experiments.

All three bacteriophages retained their activity after incubation within a temperature range between −20 °C and 40 °C (*p* < 0.05) (Figure 6a). It can be assumed that, in practice, phage storage and application refer to the temperature range of 4 °C to 40 °C. Comparable stability at a temperature not exceeding 40 °C was noted in many coliphages of therapeutic importance [40,41,42,43]. Furthermore, phages BF9 and BF17 retained the ability to infect bacteria after incubation at 50 °C (*p* < 0.05) (Figure 6). This is noteworthy because some technological processes used in phage manufacturing, e.g., spray drying, require viral activity to be maintained at extremely high temperatures often exceeding 60 °C, but some reports show that the use of temperatures of 40–45 °C may also be effective [44]. This seems to be crucial as to date there are no satisfactory methods for stabilization of bacteriophages for commercial use. 

**Figure 7:**
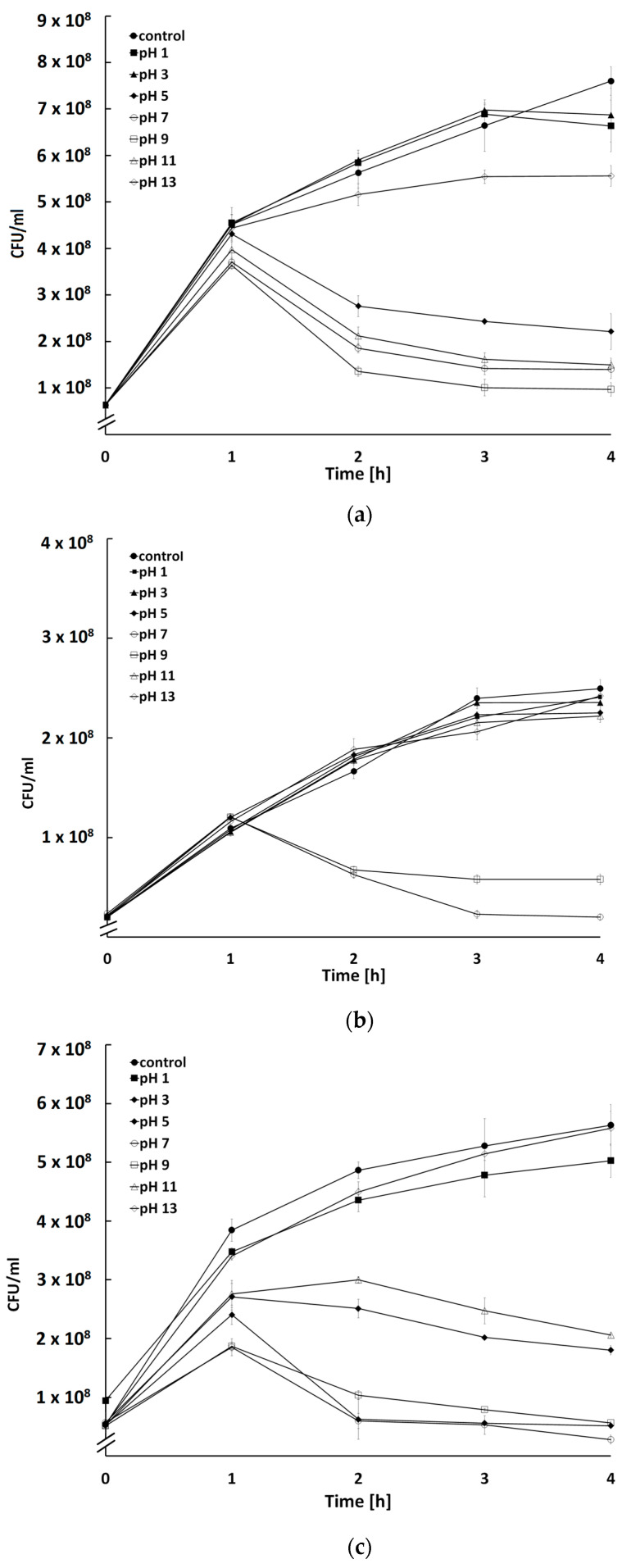
Bactericidal activity of phages BF9 (**a**), BF15 (**b**), and BF17 (**c**) pre-incubated at different pH on count of the host strain. Each data point represents the mean and standard deviation of three different experiments.

The vast majority of bacteriophages retain biological activity at neutral as well as slightly acidic and slightly alkaline pH [32,45,46,47]. In accordance, phages BF9, BF15, and BF17 maintained infectivity after incubation in the pH range of 5–9 (Figure 7). A slight decrease in BF17 viability was observed after preincubation at pH 3 and pH 11 (*p* < 0.05) (Figure 7c). Phages retaining activity at pH 1–2 have been identified extremely rarely, and the tolerance observed was most likely the result of the accumulation of irreversible mutations during low-pH incubation [48]. This can be important for the potential use of phages, because the low pH of the stomach serves as the greatest challenge to the oral administration of the phage preparation [49].

## 3. Materials and Methods

### 3.1. Bacteriophages and Bacteria

Bacteriophages and their host bacteria (Table 1) were obtained from the Collection of Microorganisms of the Department of Biotechnology and Food Microbiology, Wrocław University of Environmental and Life Sciences (Wrocław, Poland).

### 3.2. Bacteriophage Propagation

Bacteriophages BF9, BF15, and BF17 were amplified on their host bacteria (Table 1). Phages were amplified following protocols provided by Skaradzińska et al. [50]. Briefly, liquid LB medium (10 mL) was inoculated with a single colony of the respective host bacterial strain (Table 1) and incubated overnight at 37 °C with shaking (150 rpm). Next, 5 mL of bacteriophage lysate (1 × 10^4^ PFU/mL) was added to the bacterial culture. The phage-bacteria culture was incubated for 24 h at 37 °C (150 rpm). Then the culture was centrifuged (5000 rpm, t = 10 min, 20 °C) and the supernatant was filtered through a 0.22 µm syringe filter (Merck Millipore, Darmstadt, Germany). This filtrate was subsequently added to a 20 h host culture in 100 mL of liquid LB medium. The suspension was again incubated for 24 h at 37 °C with shaking (150 rpm). The culture was centrifuged and filtered through a 0.22 µm syringe filter (Merck Millipore, Darmstadt, Germany). The double-agar overlay method was used for plaque formation and determination of bacteriophage titer (PFU/mL) [51].

### 3.3. Morphology of Phage Virions and Plaques

The plaque morphology of BF9, BF15, and BF17 bacteriophages was tested on the host strains *E. coli* B19, *E. coli* B39, and *E. coli* B73, respectively. To determine the plaque size, 10-fold dilutions of virus stocks were prepared in LB. Then, 200 µL of overnight host culture was mixed with 100 µL of an appropriate dilution of phage lysate and added to 3 mL of LB with 0.3% agar. The mixture was poured onto plates containing 25 mL of LB agar. The double-layer agar plates were incubated at 37 °C for 20 h. The next day, plaque morphology was determined.

Phage preparation for electron microscopy was carried out according to a method described by Ackermann [52] and Kęsik-Szeloch et al. [53] with modifications. Briefly, a sterile, high-titer lysate was centrifuged for 60 min at 25,000× *g*. The supernatant was discarded, replaced by a smaller volume of 0.1 M ammonium acetate, and gently mixed by pipetting. A drop of washed phage suspension was deposited on formvar/carbon-supported copper grids and left to absorb for 1 min. Then, the liquid was drained off with filter paper. After that, a drop of 2% uranyl acetate was placed on a grid and drained after 1 min. This stained preparation was examined using the transmission electron microscope (TEM). The transmission electron microscope Jeol JEM-F 200 with accelerating voltage 80 kv and the TVIPS Tem Cam camera (XF-Series) were used for phage morphology studies.

### 3.4. Bacteriophage DNA Extraction, Sequencing, and Bioinformatic Analysis

Before the extraction, high-titer phage lysates obtained according to the procedure described above were treated with 10 µg/mL DNase I and 6 µg/mL RNase A (Thermo Fisher Scientific, Waltham, MA, USA) for 30 min at 37 °C. Next, 25 mM EDTA at pH 8.0 was added to inhibit nucleases and the culture was further incubated at 65 °C for 15 min. An equal volume of chloroform was added and the culture was centrifuged at 12,000 rpm for 10 min at room temperature. The genomic DNA was extracted using a High Pure Viral Nucleic Acid Large Volume Kit (Roche, Mannheim, Germany) following the manufacturer’s instructions.

Genomic DNA was sequenced using a MiSeq Illumina sequencer with a 2 × 300 bp read length (Genomed S.A., Warsaw, Poland). All sequenced phages were de novo assembled using a CLC Genomic Workbench. Topology of the phage genome has been inferred with PhageTerm software version 1.0.11 [19]. The report generated by this software is included in Supplementary Materials (Figure S1). For each bacteriophage, a single contig was generated. Putative ORFs were identified by GeneMarkS [54] and manually verified with BLAST [55] and PHASTER servers [56]. The functions of ORFs were predicted using BLASTp searches against non-redundant protein sequences (E < 10^−5^) and the UniProtKB/Swiss-Prot (UniProt Consortium) database. ORFs were screened against determinants of antimicrobial resistance using the ResFinder database [57] and for the determination of virulence factors using VFDB [58]. Putative transfer RNA (tRNA)-encoding genes were screened using ARAGORN [59].

The whole genome sequences of BF9, BF15, and BF17 phages were subjected to phylogenetic analyses using the Virus Classification and Tree building Online Resource (VICTOR) (https://ggdc.dsmz.de/victor.php, accessed on 9 January 2023). Phage sequences were obtained from the NCBI nucleotide database. All pairwise comparisons of the nucleotide sequences were conducted using the Genome-BLAST Distance Phylogeny (GBDP) method under the settings recommended for prokaryotic viruses [60]. The resulting intergenomic distances were used to infer a balanced minimum evolution tree with branch support (including 100 pseud-bootstrap replicates each) via FASTME, including subtree pruning and regrafting postprocessing for the formula D0 [61]. Phylogenetic trees were rooted at the midpoint and visualized by iTOL (https://itol.embl.de/, accessed on 11 January 2023) [62].

According to ICTV recommendations, the phage’s taxonomy was further delineated using VIRIDIC (http://rhea.icbm.uni-oldenburg.de/VIRIDIC/, accessed on 13 January 2023) [63], which calculates the virus intergenomic distance under the BLASTn default settings. The dataset used for VIRIDIC consisted of phages and their closest relatives based on BLASTn and whole-genome phylogeny.

The comparison between phages, their closest relative, and type species of respective genus was performed using EasyFig v2.2.2 [64] and CoreGenes5.0 with a threshold of 75% [65].

### 3.5. Bacterial Host Reduction at Different MOIs

The ability of phages to inactivate bacterial hosts (Table 1) was evaluated at different phage/bacteria ratios (MOI values ranging from 0.00001 to 10) over time with a modified method of Abdelrahman et al. [66] and Duarte et al. [67]. A total of 180 µL of host culture was diluted to an optical density (OD_600_) of 0.2 corresponding to a bacterial count of approximately 1 × 10^8^ CFU/mL and loaded into wells of flat-bottomed 48-well microtiter plates with a lid and inoculated with 20 µL of phage lysate at different MOIs. Cultures without phage suspension were used as controls. The plates were incubated at 37 °C with shaking (150 rpm) for 12 h and bacterial growth was measured every hour using a Spark Multimode Microplate Reader (Tecan Trading AG, Männedorf, Switzerland). The OD_600_ values were converted to CFU/mL using additional standard curves representing the relationship between bacterial count and the optical density of the culture.

### 3.6. Influence of Temperature on Activity of Phages

For studies of phage thermal stability, 1 mL of phage preparation at a titer of 1 × 10^9^ PFU/mL was placed into each of the seven test tubes and preincubated at −20 °C, 4 °C, 20 °C, 30 °C, 40 °C, 50 °C, and 60 °C for 20 h. Next, 20 µL of phage suspensions previously incubated at different temperatures were added to 180 µL of host culture diluted to OD_600_ = 0.2 (approx. 1 × 10^8^ CFU/mL) and loaded into wells of flat-bottomed 48-well microtiter plates with a lid. Cultures without phage suspension were used as a control. The plates were incubated at 37 °C with shaking for 4 h and bacterial growth was measured every half hour using a Spark Multimode Microplate Reader (Tecan Trading AG, Männedorf, Switzerland). The OD_600_ values were converted to CFU/mL using additional standard curves representing the relationship between bacterial count and the optical density of the culture.

### 3.7. Influence of pH on Activity of Phages

The stability of phages was tested a wide range of pH, 1–13. A total of 10 mL of bacteriophage preparations at a titer of 1 × 10^9^ PFU/mL were placed into each of the seven test tubes and the pH was adjusted to 1, 3, 5, 7, 9, 11, and 13 with 1 M NaOH and 1 M HCl. As pH was adjusted in non-sterile conditions, all preparations were subsequently filtered with a syringe filter with a pore size of 0.22 µm (Merck Millipore, Darmstadt, Germany). Next, 20 µL of phage suspensions previously incubated at different pH were added to 180 µL of host culture diluted to OD_600_ = 0.2 (approx. 1 × 10^8^ CFU/mL) and loaded into wells of flat-bottomed 48-well microtiter plates with a lid. Cultures without phage suspension were used as a control. The plates were incubated at 37 °C with shaking for 4 h and bacterial growth was measured every half hour using a Spark Multimode Microplate Reader (Tecan Trading AG, Männedorf, Switzerland). The OD_600_ values were converted to CFU/mL using additional standard curves representing the relationship between bacterial count and the optical density of the culture.

### 3.8. Statistical Analysis

All experiments were repeated at least three times. Statistical analysis was performed using STATISTICA v. 13.3 (TIBCO Software Inc., Palo Alto, CA, USA). One-way ANOVA with repeated measures was used to analyze the differences between the growth curves of host cultures in the presence and absence of the phages during the sampling period. The differences were considered significant at a *p*-value of <0.05.

## 4. Conclusions

We previously isolated bacteriophages with a broad activity spectrum against ESBL/AmpC *E. coli*. However, the performed studies lacked detailed characteristics of phages that would confirm the safety of phages. In this work, we found that bacteriophages BF9, BF15, and BF17 differ significantly and all display favorable properties for successful outcomes of future applications. Detailed genomic annotation excluded the presence of toxin-coding genes, antibiotic resistance determinants, and other bacterial virulence factors. The results described herein support the utility of BF9, BF15, and BF17 as biocontrol agents to reduce the prevalence of antibiotic-resistant bacteria originating from food-producing animals.

However, comprehensive studies are needed to understand the impact of bacteriophages on the transmission of antimicrobial-resistant bacteria into the environment and whether phages would minimize the maintenance of bacteria and/or resistant genes outside farming environments.

## Figures and Tables

**Table 1:** Bacteriophages and bacteria used in the study.

Bacteriophage	Bacterial Isolates	Bacterial Sources	Reference
BF9	*Escherichia coli* B19	pig manure	[16]
BF15	*Escherichia coli* B39	pig manure	
BF17	*Escherichia coli* B73	pig manure	

## Data Availability

The annotated complete genomes of BF9, BF15, and BF17 were deposited in GenBank under accession numbers MW822006, MW822007, and MW822008.

## Supplementary Materials

The following are available online at https://www.mdpi.com/article/10.3390/ijms24065696/s1.
