# Supplementary material for: Characterization and Comparative Genomic Analysis of Three Virulent E. coli Bacteriophages with the Potential to Reduce Antibiotic-Resistant Bacteria in the Environment
Source: Int J Mol Sci. 2023 Mar 16;24(6):5696. doi: 10.3390/ijms24065696 (PMC10059673; doi:10.3390/ijms24065696)
Supplement: Supplementary file 1 [file ijms-24-05696-s001.zip › Supplementary Figure S1.pdf]

(a)

BF9_new PhageTerm Analysis

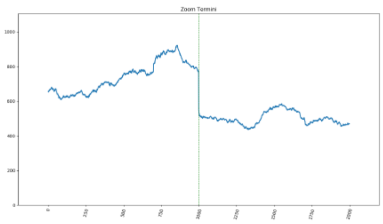

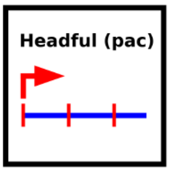

PhageTerm Method

Ends	Left (red)	Right (green)	Permuted	Orientation	Class	Type
Redundant	Distributed	28168	Yes	Reverse	Headful (pac)	P1

Strand	Location	T	pvalue
+	4381	0.04	1.00e+00
	37563	0.03	1.00e+00
	35182	0.03	1.00e+00
	15890	0.03	1.00e+00
	16012	0.03	1.00e+00
-	28168	0.53	8.06e-65
	38030	0.04	3.27e-02
	35314	0.03	1.00e+00
	6351	0.03	1.00e+00
	13386	0.03	1.00e+00

T (Start. Pos. Cov. / Whole Cov.)

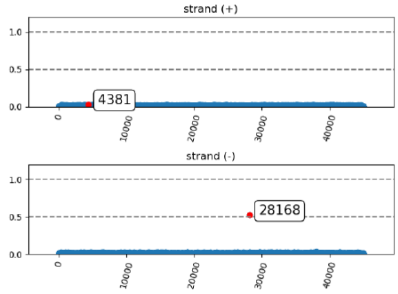

Li's Method

Packaging	Termini	Forward	Reverse	Orientation
PAC	Fixed	Multiple-Pref. Term.	Obvious Termini	Reverse

(b)

BF15 PhageTerm Analysis

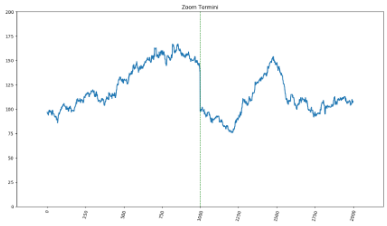

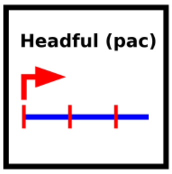

PhageTerm Method

Ends	Left (red)	Right (green)	Permuted	Orientation	Class	Type
Redundant	Distributed	31929	Yes	Reverse	Headful (pac)	P1

Strand	Location	T	pvalue
+	89661	0.22	7.05e-03
	106113	0.21	8.35e-04
	64992	0.18	9.41e-02
	146351	0.16	1.00e+00
	155006	0.15	2.13e-01
-	31929	0.38	2.93e-08
	24329	0.19	1.00e+00
	9398	0.17	1.00e+00
	44358	0.17	3.97e-01
	36015	0.16	1.00e+00

T (Start. Pos. Cov. / Whole Cov.)

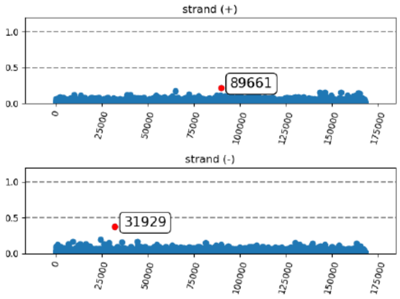

(c)

BF17 PhageTerm Analysis

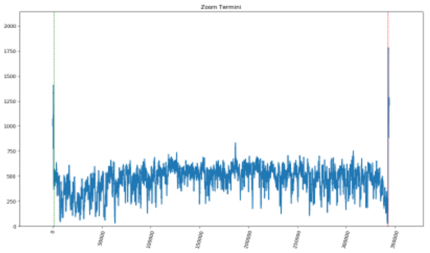

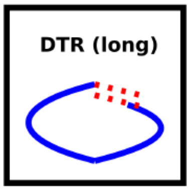

PhageTerm Method

Ends	Left (red)	Right (green)	Permuted	Orientation	Class	Type
Redundant	343150	838	No	NA	DTR (long)	T5

**Direct Terminal Repeats: 20590 bp*

Strand	Location	T	pvalue
+	343150	0.76	0.00e+00
	328452	0.04	1.00e+00
	256072	0.04	1.00e+00
	178079	0.04	1.00e+00
	307012	0.04	1.00e+00
-	838	0.61	3.89e-159
	95609	0.04	1.00e+00
	279233	0.04	1.00e+00
	218577	0.04	5.38e-01
	104749	0.03	1.00e+00

T (Start. Pos. Cov. / Whole Cov.)

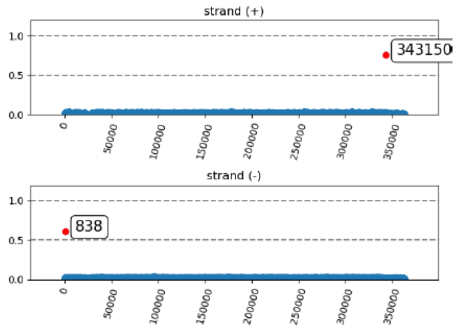

Figure S1. PhageTerm analysis of the BF9 (a), BF15 (b), and BF17 (c) sequence read data demonstrating the genome packaging mechanism.
