# Supplementary material for: Characterization and Comparative Genomic Analysis of Three Virulent E. coli Bacteriophages with the Potential to Reduce Antibiotic-Resistant Bacteria in the Environment
Source: Int J Mol Sci. 2023 Mar 16;24(6):5696. doi: 10.3390/ijms24065696 (PMC10059673; doi:10.3390/ijms24065696)
Supplement: Supplementary file 1 [file ijms-24-05696-s001.zip › Supplementary Table S1.pdf]

Table S1. The overview of general features of bacteriophages genomes.

Phage	GeneBank Accession	Host strain	Genome length (bp)	G+C content (%)	ORFs	tRNA genes	Lifestyle	Taxonomy	Core genes shared with representative of genera
BF9	MW822006	*E. coli* B19	44,963	54,5	58	0	lytic	*Dhillonvirus*	47 HK578 (JQ086375)
BF15	MW822007	*E. coli* B39	167,801	35,4	267	8	lytic	*Tequatrovirus*	241 T4 (NC_000866)
BF17	MW822008	*E. coli* B73	383,493	35,4	660	6	lytic	*Asteriusvirus*	569 PBECO4 (KC295538)
