# Supplementary material for: Characterization and Comparative Genomic Analysis of Three Virulent E. coli Bacteriophages with the Potential to Reduce Antibiotic-Resistant Bacteria in the Environment
Source: Int J Mol Sci. 2023 Mar 16;24(6):5696. doi: 10.3390/ijms24065696 (PMC10059673; doi:10.3390/ijms24065696)
Supplement: Supplementary file 1 [file ijms-24-05696-s001.zip › Supplementary Table S3.pdf]

Table S3 List of genes shared by phages BF9, BF15, and BF17 with the most representative phages of the genus performed with CoreGenes5.0

**Escherichia phage BF9** *MW822006.1*	**Escherichia phage HK578** *JQ086375.1*
PI:*QXL91276.1*<br>Product:putative lipoprotein	PI:*AFH20529.1*<br>Product:hypothetical protein
PI:*QXL91277.1*<br>Product:hypothetical protein	PI:*AFH20528.1*<br>Product:hypothetical protein
PI:*QXL91278.1*<br>Product:putative tail fiber tip protein	PI:*AFH20527.1*<br>Product:bacteriophage host specificity protein
PI:*QXL91279.1*<br>Product:tail assembly protein	PI:*AFH20526.1*<br>Product:tail assembly protein
PI:*QXL91280.1*<br>Product:minor tail protein	PI:*AFH20525.1*<br>Product:tail component protein
PI:*QXL91281.1*<br>Product:minor tail protein	PI:*AFH20524.1*<br>Product:minor tail protein
PI:*QXL91282.1*<br>Product:tail length tape measure protein	PI:*AFH20522.1*<br>Product:tail length tape measure protein
PI:*QXL91283.1*<br>Product:hypothetical protein	PI:*AFH20521.1*<br>Product:hypothetical protein
PI:*QXL91284.1*<br>Product:hypothetical protein	PI:*AFH20520.1*<br>Product:hypothetical protein
PI:*QXL91285.1*<br>Product:major tail protein	PI:*AFH20519.1*<br>Product:tail protein
PI:*QXL91286.1*<br>Product:hypothetical protein	PI:*AFH20518.1*<br>Product:hypothetical protein
PI:*QXL91287.1*<br>Product:tail protein	PI:*AFH20517.1*<br>Product:hypothetical protein
PI:*QXL91288.1*<br>Product:hypothetical protein	PI:*AFH20516.1*<br>Product:hypothetical protein
PI:*QXL91289.1*<br>Product:hypothetical protein	PI:*AFH20514.1*<br>Product:hypothetical protein
PI:*QXL91290.1*<br>Product:major head protein	PI:*AFH20513.1*<br>Product:major head protein
PI:*QXL91291.1*<br>Product:hypothetical protein	PI:*AFH20512.1*<br>Product:hypothetical protein
PI:*QXL91293.1*<br>Product:hypothetical protein	PI:*AFH20510.1*<br>Product:hypothetical protein
PI:*QXL91294.1*<br>Product:head morphogenesis protein	PI:*AFH20509.1*<br>Product:head morphogenesis protein
PI:*QXL91295.1*<br>Product:structural protein	PI:*AFH20508.1*<br>Product:hypothetical protein
PI:*QXL91296.1*<br>Product:terminase large subunit	PI:*AFH20507.1*<br>Product:terminase large subunit
PI:*QXL91297.1*<br>Product:terminase small subunit	PI:*AFH20506.1*<br>Product:terminase small subunit

PI:*QXL91298.1* Product:hypothetical protein	PI:*AFH20565.1* Product:hypothetical protein
PI:*QXL91299.1* Product:HTH luxR-type domain-containing protein	PI:*AFH20564.1* Product:hypothetical protein
PI:*QXL91301.1* Product:lysozyme	PI:*AFH20562.1* Product:lysozyme
PI:*QXL91302.1* Product:hypothetical protein	PI:*AFH20561.1* Product:hypothetical protein
PI:*QXL91303.1* Product:hypothetical protein	PI:*AFH20560.1* Product:hypothetical protein
PI:*QXL91304.1* Product:hypothetical protein	PI:*AFH20559.1* Product:hypothetical protein
PI:*QXL91306.1* Product:hypothetical protein	PI:*AFH20554.1* Product:hypothetical protein
PI:*QXL91308.1* Product:DNA helicase	PI:*AFH20551.1* Product:hypothetical protein
PI:*QXL91309.1* Product:hypothetical protein	PI:*AFH20550.1* Product:hypothetical protein
PI:*QXL91310.1* Product:hypothetical protein	PI:*AFH20549.1* Product:hypothetical protein
PI:*QXL91311.1* Product:hypothetical protein	PI:*AFH20548.1* Product:hypothetical protein
PI:*QXL91313.1* Product:hypothetical protein	PI:*AFH20546.1* Product:hypothetical protein
PI:*QXL91315.1* Product:hypothetical protein	PI:*AFH20545.1* Product:hypothetical protein
PI:*QXL91316.1* Product:hypothetical protein	PI:*AFH20544.1* Product:hypothetical protein
PI:*QXL91317.1* Product:DNA polymerase	PI:*AFH20543.1* Product:hypothetical protein
PI:*QXL91319.1* Product:VRR-NUC domain-containing protein	PI:*AFH20541.1* Product:hypothetical protein
PI:*QXL91320.1* Product:hypothetical protein	PI:*AFH20540.1* Product:hypothetical protein
PI:*QXL91321.1* Product:hypothetical protein	PI:*AFH20539.1* Product:hypothetical protein
PI:*QXL91322.1* Product:DNA helicase	PI:*AFH20538.1* Product:hypothetical protein
PI:*QXL91323.1* Product:hypothetical protein	PI:*AFH20537.1* Product:hypothetical protein
PI:*QXL91324.1* Product:HNH endonuclease	PI:*AFH20536.1* Product:hypothetical protein
PI:*QXL91327.1* Product:DNA N-6-adenine methyltransferase	PI:*AFH20535.1* Product:DNA N-6-adenine-methyltransferase
PI:*QXL91328.1* Product:hypothetical protein	PI:*AFH20534.1* Product:hypothetical protein
PI:*QXL91329.1* Product:hypothetical protein	PI:*AFH20533.1* Product:hypothetical protein

PI:*QXL91330.1*<br>Product:hypothetical protein	PI:*AFH20532.1*<br>Product:hypothetical protein
PI:*QXL91331.1*<br>Product:hypothetical protein	PI:*AFH20531.1*<br>Product:hypothetical protein

**Escherichia phage BF15**<br>***MW822007.1***	**Escherichia phage T4**<br>***NC_000866.4***
PI:*QXN75718.1*<br>Product:exonuclease A	PI:*NP_049629.1*<br>Product:3'-5' exoribonuclease
PI:*QXN75719.1*<br>Product:exonuclease	PI:*NP_049630.1*<br>Product:DexA.1 hypothetical protein
PI:*QXN75720.1*<br>Product:exonuclease	PI:*NP_049631.1*<br>Product:dextranase
PI:*QXN75721.1*<br>Product:DNA helicase	PI:*NP_049632.1*<br>Product:AAA family ATPase
PI:*QXN75722.1*<br>Product:hypothetical protein	PI:*NP_049633.1*<br>Product:Dda.1 hypothetical protein
PI:*QXN75723.1*<br>Product:hypothetical protein	PI:*NP_049634.1*<br>Product:Srd anti-sigma factor
PI:*QXN75724.1*<br>Product:NAD--protein ADP-ribosyltransferase	PI:*NP_049635.1*<br>Product:ModA RNA polymerase ADP-ribosylase
PI:*QXN75725.1*<br>Product:NAD--protein ADP-ribosyltransferase	PI:*NP_049636.1*<br>Product:ModB ADP-ribosylase
PI:*QXN75726.1*<br>Product:hypothetical protein	PI:*NP_049637.1*<br>Product:hypothetical protein
PI:*QXN75727.1*<br>Product:hypothetical protein	PI:*NP_049638.1*<br>Product:ModA.3 hypothetical protein
PI:*QXN75728.1*<br>Product:hypothetical protein	PI:*NP_049639.1*<br>Product:ModA.4 hypothetical protein
PI:*QXN75729.1*<br>Product:hypothetical protein	PI:*NP_049640.1*<br>Product:Srh transcription modulator
PI:*QXN75730.1*<br>Product:transcription modulator under heat shock	PI:*NP_049641.1*<br>Product:Mrh transcription modulator under heat shock
PI:*QXN75731.1*<br>Product:hypothetical protein	PI:*NP_049642.1*<br>Product:Mrh.1 hypothetical protein
PI:*QXN75732.1*<br>Product:hypothetical protein	PI:*NP_049643.1*<br>Product:Mrh.2 hypothetical protein
PI:*QXN75733.1*<br>Product:small outer capsid protein	PI:*NP_049644.1*<br>Product:Soc small outer capsid protein
PI:*QXN75734.1*<br>Product:dCTP pyrophosphatase	PI:*NP_049646.1*<br>Product:dUTP diphosphatase
PI:*QXN75735.1*<br>Product:DNA methyltransferase	PI:*NP_049647.1*<br>Product:Dam family site-specific
PI:*QXN75738.1*<br>Product:DNA primase	PI:*NP_049648.1*<br>Product:DNA primase subunit
PI:*QXN75739.1*<br>Product:hypothetical protein	PI:*NP_049649.1*<br>Product:gp61.1 hypothetical protein

PI:*QXN75741.1*<br>Product:spackle periplasmic protein	PI:*NP_049651.1*<br>Product:sp spackle periplasmic protein
PI:*QXN75742.1*<br>Product:hypothetical protein	PI:*NP_049652.1*<br>Product:gp61.4 hypothetical protein
PI:*QXN75743.1*<br>Product:hypothetical protein	PI:*NP_049653.1*<br>Product:Dmd discriminator of mRNA degradation
PI:*QXN75744.1*<br>Product:DNA primase-helicase subunit	PI:*NP_049654.1*<br>Product:AAA family ATPase
PI:*QXN75745.1*<br>Product:head vertiex assembly chaperone	PI:*NP_049655.1*<br>Product:gp40 head vertex assembly chaperone
PI:*QXN75746.1*<br>Product:recombination protein	PI:*NP_049656.2*<br>Product:recombinase RecA
PI:*QXN75749.1*<br>Product:thymidylate synthase	PI:*NP_049659.1*<br>Product:thymidylate synthase
PI:*QXN75750.1*<br>Product:immunity to superinfection protein	PI:*NP_049660.1*<br>Product:superinfection immunity protein
PI:*QXN75751.1*<br>Product:immunity protein	PI:*NP_049661.1*<br>Product:iduronate sulfatase
PI:*QXN75752.1*<br>Product:DNA polymerase	PI:*NP_049662.1*<br>Product:DNA polymerase
PI:*QXN75753.1*<br>Product:translation repressor protein	PI:*NP_049663.1*<br>Product:translational repressor RegA
PI:*QXN75754.1*<br>Product:sliding-clamp-loader subunit	PI:*NP_049664.1*<br>Product:DNA polymerase clamp loader subunit A
PI:*QXN75755.1*<br>Product:sliding-clamp-loader subunit	PI:*NP_049665.1*<br>Product:AAA family ATPase
PI:*QXN75756.1*<br>Product:DNA polymerase clamp	PI:*NP_049666.1*<br>Product:sliding clamp
PI:*QXN75757.1*<br>Product:RNA polymerase-binding protein	PI:*NP_049667.1*<br>Product:RNA polymerase-binding protein RpbA
PI:*QXN75758.1*<br>Product:hypothetical protein	PI:*NP_049668.1*<br>Product:protein GP45.2
PI:*QXN75759.1*<br>Product:recombination exonuclease subunit 2	PI:*NP_049669.1*<br>Product:AAA family ATPase
PI:*QXN75760.1*<br>Product:hypothetical protein	PI:*NP_049670.1*<br>Product:DUF5487 family protein
PI:*QXN75761.1*<br>Product:hypothetical protein	PI:*NP_049671.1*<br>Product:gp46.2 hypothetical protein
PI:*QXN75762.1*<br>Product:recombination exonuclease subunit 1	PI:*NP_049672.1*<br>Product:metallophosphoesterase
PI:*QXN75763.1*<br>Product:membrane protein	PI:*NP_813808.1*<br>Product:gp47.1 hypothetical predicted membrane protein
PI:*QXN75764.1*<br>Product:DNA alpha-glucosyltransferase	PI:*NP_049673.1*<br>Product:a-gt alpha glucosyl transferase
PI:*QXN75765.1*<br>Product:hypothetical protein	PI:*NP_049675.1*<br>Product:a-gt.2 hypothetical protein

PI:*QXN75766.1*<br>Product:hypothetical protein	PI:*NP_049676.1*<br>Product:a-gt.3 hypothetical protein
PI:*QXN75767.1*<br>Product:hypothetical protein	PI:*NP_049677.1*<br>Product:a-gt.4 family protein
PI:*QXN75768.1*<br>Product:hypothetical protein	PI:*NP_049678.1*<br>Product:a-gt.5 hypothetical protein
PI:*QXN75769.1*<br>Product:RNA polymerase sigma factor	PI:*NP_049679.1*<br>Product:RNA polymerase sigma factor
PI:*QXN75771.1*<br>Product:hypothetical protein	PI:*NP_049680.1*<br>Product:gp55.1 hypothetical protein
PI:*QXN75772.1*<br>Product:hypothetical protein	PI:*NP_049681.1*<br>Product:gp55.2 hypothetical protein
PI:*QXN75773.1*<br>Product:hypothetical protein	PI:*NP_049682.1*<br>Product:gp55.3 hypothetical protein
PI:*QXN75774.1*<br>Product:hypothetical protein	PI:*NP_049683.1*<br>Product:gp55.4 conserved hypothetical protein
PI:*QXN75775.1*<br>Product:hypothetical protein	PI:*NP_049684.1*<br>Product:gp55.5 conserved protein of unknown function
PI:*QXN75776.1*<br>Product:hypothetical protein	PI:*NP_049685.1*<br>Product:gp55.6 conserved hypothetical protein
PI:*QXN75777.1*<br>Product:glutaredoxin	PI:*NP_049686.1*<br>Product:NrdH glutaredoxin
PI:*QXN75778.1*<br>Product:hypothetical protein	PI:*NP_049687.1*<br>Product:gp55.8 conserved hypothetical predicted membrane
PI:*QXN75780.1*<br>Product:anaerobic ribonucleoside-triphosphate	PI:*NP_049688.1*<br>Product:anaerobic ribonucleoside-triphosphate reductase
PI:*QXN75782.1*<br>Product:anaerobic ribonucleoside-triphosphate reductase	PI:*NP_049690.1*<br>Product:anaerobic ribonucleoside-triphosphate reductase
PI:*QXN75783.1*<br>Product:recombination endonuclease VII	PI:*NP_049692.1*<br>Product:recombination endonuclease VII
PI:*QXN75784.1*<br>Product:peptidase inhibitor	PI:*NP_049694.1*<br>Product:peptidase
PI:*QXN75785.1*<br>Product:hypothetical protein	PI:*NP_049695.1*<br>Product:gp49.1 conserved protein of unknown function
PI:*QXN75788.1*<br>Product:thioredoxin	PI:*NP_049698.1*<br>Product:NrdC thioredoxin
PI:*QXN75789.1*<br>Product:thioredoxin	PI:*NP_049699.1*<br>Product:NrdC.1 conserved hypothetical protein
PI:*QXN75790.1*<br>Product:hypothetical protein	PI:*NP_049700.1*<br>Product:NrdC.2 conserved hypothetical protein
PI:*QXN75791.1*<br>Product:hypothetical protein	PI:*NP_049701.1*<br>Product:NrdC.3 conserved hypothetical protein
PI:*QXN75792.1*<br>Product:thioredoxin	PI:*NP_049702.1*<br>Product:NrdC.4 conserved hypothetical protein
PI:*QXN75793.1*<br>Product:thioredoxin	PI:*NP_049703.1*<br>Product:NrdC.5 conserved hypothetical protein

PI:*QXN75794.1*<br>Product:thioredoxin	PI:*NP_049704.1*<br>Product:NrdC.6 conserved hypothetical protein
PI:*QXN75795.1*<br>Product:thioredoxin	PI:*NP_049705.1*<br>Product:NrdC.7 conserved hypothetical
PI:*QXN75796.1*<br>Product:thioredoxin	PI:*NP_049706.1*<br>Product:NrdC.8 conserved hypothetical protein
PI:*QXN75797.1*<br>Product:hypothetical protein	PI:*NP_049707.1*<br>Product:NrdC.9 conserved hypothetical protein
PI:*QXN75799.1*<br>Product:AAA domain-containing protein	PI:*NP_049708.1*<br>Product:NrdC.10 conserved hypothetical protein
PI:*QXN75800.1*<br>Product:hypothetical protein	PI:*NP_049709.1*<br>Product:nucleotidyltransferase domain-containing
PI:*QXN75803.1*<br>Product:hypothetical protein	PI:*NP_049711.1*<br>Product:MobD.1 conserved hypothetical protein
PI:*QXN75804.1*<br>Product:hypothetical protein	PI:*NP_049712.1*<br>Product:MobD.2 conserved hypothetical protein
PI:*QXN75805.1*<br>Product:hypothetical protein	PI:*NP_813809.1*<br>Product:MobD.2a hypothetical protein
PI:*QXN75806.1*<br>Product:hypothetical protein	PI:*NP_049713.1*<br>Product:MobD.3 hypothetical protein
PI:*QXN75807.1*<br>Product:hypothetical protein	PI:*NP_049714.1*<br>Product:molybdopterin-guanine dinucleotide biosynthesis
PI:*QXN75808.1*<br>Product:hypothetical protein	PI:*NP_049715.1*<br>Product:MobD.5 hypothetical protein
PI:*QXN75809.1*<br>Product:hypothetical protein	PI:*NP_049716.1*<br>Product:DUF5856 family protein
PI:*QXN75810.1*<br>Product:antiholin	PI:*NP_049717.1*<br>Product:rI lysis inhibition regulator
PI:*QXN75811.1*<br>Product:hypothetical protein	PI:*NP_049718.1*<br>Product:rI.1 conserved hypothetical protein
PI:*QXN75812.1*<br>Product:thymidine kinase	PI:*NP_049719.1*<br>Product:thymidine kinase
PI:*QXN75813.1*<br>Product:hypothetical protein	PI:*NP_049720.1*<br>Product:Tk.1 conserved hypothetical protein
PI:*QXN75814.1*<br>Product:hypothetical protein	PI:*NP_049721.1*<br>Product:Tk.2 conserved hypothetical protein
PI:*QXN75815.1*<br>Product:hypothetical protein	PI:*NP_049722.1*<br>Product:Tk.2 conserved hypothetical protein
PI:*QXN75816.1*<br>Product:hypothetical protein	PI:*NP_049723.1*<br>Product:macro domain-containing protein
PI:*QXN75817.1*<br>Product:valyl--tRNA ligase modifier	PI:*NP_049724.1*<br>Product:Vs valyl-tRNA synthetase modifier
PI:*QXN75818.1*<br>Product:SLT domain-containing protein	PI:*NP_049725.1*<br>Product:transglycosylase SLT domain-containing protein
PI:*QXN75819.1*<br>Product:endoribonuclease	PI:*NP_049726.1*<br>Product:site-specific RNA endonuclease

PI:*QXN75820.1*<br>Product:hypothetical protein	PI:*NP_049727.1*<br>Product:Vs.3 conserved hypothetical protein
PI:*QXN75821.1*<br>Product:hypothetical protein	PI:*NP_049728.1*<br>Product:Vs.4 conserved hypothetical protein
PI:*QXN75823.1*<br>Product:pyruvate formate-lyase	PI:*NP_049730.1*<br>Product:autonomous glycyl radical cofactor GrcA
PI:*QXN75824.1*<br>Product:hypothetical protein	PI:*NP_049731.1*<br>Product:Vs.7 conserved hypothetical protein
PI:*QXN75825.1*<br>Product:hypothetical protein	PI:*NP_049732.1*<br>Product:Vs.8 conserved hypothetical protein
PI:*QXN75826.1*<br>Product:hypothetical protein	PI:*NP_049734.1*<br>Product:IpII internal head protein
PI:*QXN75827.1*<br>Product:hypothetical protein	PI:*NP_049735.1*<br>Product:IpIII internal head protein
PI:*QXN75830.1*<br>Product:baseplate hub subunit and tail lysozyme	PI:*NP_049736.1*<br>Product:glycoside hydrolase family protein
PI:*QXN75831.1*<br>Product:nudix hydrolase	PI:*NP_049737.1*<br>Product:NUDIX hydrolase
PI:*QXN75832.1*<br>Product:hypothetical protein	PI:*NP_049738.1*<br>Product:e.2 conserved hypothetical
PI:*QXN75833.1*<br>Product:hypothetical protein	PI:*NP_049739.1*<br>Product:sulfurtransferase
PI:*QXN75834.1*<br>Product:hypothetical protein	PI:*NP_049740.1*<br>Product:sulfurtransferase
PI:*QXN75835.1*<br>Product:hypothetical protein	PI:*NP_049741.1*<br>Product:e.5 conserved hypothetical protein
PI:*QXN75836.1*<br>Product:hypothetical protein	PI:*NP_049742.1*<br>Product:e.6 conserved hypothetical protein
PI:*QXN75837.1*<br>Product:hypothetical protein	PI:*NP_049743.1*<br>Product:e.7 hypothetical protein
PI:*QXN75838.1*<br>Product:hypothetical protein	PI:*NP_049744.1*<br>Product:e.8 conserved hypothetical protein
PI:*QXN75839.1*<br>Product:group I intron endonuclease	PI:*NP_049745.2*<br>Product:GIY-YIG nuclease family protein
PI:*QXN75840.1*<br>Product:hypothetical protein	PI:*NP_049746.1*<br>Product:Trna.2 conserved hypothetical protein
PI:*QXN75841.1*<br>Product:hypothetical protein	PI:*NP_049747.1*<br>Product:Trna.3 conserved hypothetical protein
PI:*QXN75842.1*<br>Product:hypothetical protein	PI:*NP_049748.1*<br>Product:Trna.4 conserved hypothetical predicted membrane
PI:*QXN75845.1*<br>Product:hypothetical protein	PI:*NP_049750.1*<br>Product:hypothetical protein
PI:*QXN75846.1*<br>Product:tail fiber assembly protein	PI:*NP_049751.1*<br>Product:gp57A chaperone for tail fiber formation
PI:*QXN75847.1*<br>Product:deoxynucleotide monophosphate kinase	PI:*NP_049752.1*<br>Product:deoxynucleoside monophosphate kinase

PI:*QXN75848.1*<br>Product:tail completion and sheath stabilizer protein	PI:*NP_049753.1*<br>Product:tail protein
PI:*QXN75849.1*<br>Product:DNA end protector protein	PI:*NP_049754.1*<br>Product:DNA end protector protein
PI:*QXN75850.1*<br>Product:head completion protein	PI:*NP_049755.1*<br>Product:head completion protein
PI:*QXN75851.1*<br>Product:baseplate wedge protein	PI:*NP_049756.1*<br>Product:baseplate wedge protein 53
PI:*QXN75852.1*<br>Product:baseplate hub subunit and tail lysozyme	PI:*NP_049757.1*<br>Product:glycoside hydrolase family protein
PI:*QXN75853.1*<br>Product:hypothetical protein	PI:*NP_049760.1*<br>Product:hypothetical protein
PI:*QXN75854.1*<br>Product:hypothetical protein	PI:*NP_049763.1*<br>Product:PAAR domain-containing protein
PI:*QXN75855.1*<br>Product:baseplate wedge protein	PI:*NP_049764.1*<br>Product:baseplate wedge subunit
PI:*QXN75856.1*<br>Product:baseplate wedge protein	PI:*NP_049765.1*<br>Product:baseplate wedge subunit
PI:*QXN75857.1*<br>Product:baseplate wedge protein	PI:*NP_049766.1*<br>Product:baseplate wedge subunit
PI:*QXN75858.1*<br>Product:baseplate wedge tail-fiber connector	PI:*NP_049767.1*<br>Product:baseplate wedge tail fiber connector
PI:*QXN75859.1*<br>Product:baseplate wedge protein	PI:*NP_049768.1*<br>Product:tail protein
PI:*QXN75860.1*<br>Product:baseplate wedge completion tail pin	PI:*NP_049769.1*<br>Product:tail protein
PI:*QXN75861.1*<br>Product:short tail fiber protein	PI:*NP_049770.1*<br>Product:tail fiber protein
PI:*QXN75862.1*<br>Product:fibritin neck whiskers protein	PI:*NP_049771.1*<br>Product:neck protein fibritin
PI:*QXN75863.1*<br>Product:neck protein	PI:*NP_049772.1*<br>Product:putative neck protein
PI:*QXN75864.1*<br>Product:neck protein	PI:*NP_049773.1*<br>Product:neck protein
PI:*QXN75865.1*<br>Product:tail sheath stabilization protein	PI:*NP_049774.1*<br>Product:tail sheath stabilizer and completion protein
PI:*QXN75866.1*<br>Product:terminase	PI:*NP_049775.1*<br>Product:small terminase protein
PI:*QXN75867.1*<br>Product:terminase	PI:*NP_049776.1*<br>Product:terminase family protein
PI:*QXN75867.1*<br>Product:terminase	PI:*NP_049778.1*<br>Product:large terminase protein
PI:*QXN75867.1*<br>Product:terminase	PI:*NP_049779.1*<br>Product:large terminase protein
PI:*QXN75867.1*<br>Product:terminase	PI:*NP_049777.1*<br>Product:large terminase protein

PI:*QXN75868.1* Product:tail sheath protein	PI:*NP_049780.1* Product:tail sheath subtilisin-like domain-containing
PI:*QXN75869.1* Product:tail fiber protein	PI:*NP_049781.1* Product:tail protein
PI:*QXN75870.1* Product:portal protein	PI:*NP_049782.1* Product:portal protein
PI:*QXN75871.1* Product:prohead core protein	PI:*NP_049783.1* Product:prohead core protein
PI:*QXN75872.1* Product:prohead core protein	PI:*NP_049784.1* Product:prohead core protein
PI:*QXN75873.1* Product:prohead core scaffold protein and protease	PI:*NP_049785.1* Product:prohead core scaffolding protein and protease
PI:*QXN75874.1* Product:prohead core protein	PI:*NP_049786.1* Product:prohead core protein
PI:*QXN75875.1* Product:major capsid protein	PI:*NP_049787.1* Product:capsid protein
PI:*QXN75876.1* Product:capsid vertex protein	PI:*NP_049789.1* Product:capsid vertex protein
PI:*QXN75877.1* Product:RNA ligase	PI:*NP_049790.1* Product:RNA ligase
PI:*QXN75878.1* Product:hypothetical protein	PI:*NP_049791.1* Product:gp24.2 conserved hypothetical protein
PI:*QXN75879.1* Product:hypothetical protein	PI:*NP_049792.1* Product:gp24.3 conserved hypothetical protein
PI:*QXN75880.1* Product:highly immunogenic outer capsid protein	PI:*NP_049793.1* Product:PKD domain-containing protein
PI:*QXN75881.1* Product:minor capsid protein	PI:*NP_049794.1* Product:Inh inhibitor of prohead protease gp21
PI:*QXN75882.1* Product:RNA-DNA + DNA-DNA helicase	PI:*NP_049796.1* Product:UvsW helicase
PI:*QXN75883.1* Product:homing endonuclease	PI:*NP_049844.1* Product:MobE homing endonuclease
PI:*QXN75885.1* Product:hypothetical protein	PI:*NP_049797.1* Product:DUF2685 domain-containing protein
PI:*QXN75886.1* Product:hypothetical protein	PI:*NP_049798.1* Product:UvsY.-1 conserved hypothetical protein
PI:*QXN75887.1* Product:ssDNA-binding protein	PI:*NP_049799.2* Product:recombination mediator protein UvsY
PI:*QXN75888.1* Product:baseplate wedge subunit	PI:*NP_049800.1* Product:GPW/gp25 family protein
PI:*QXN75889.1* Product:baseplate hub subunit	PI:*NP_049801.1* Product:gp26 baseplate hub subunit
PI:*QXN75890.1* Product:baseplate hub assembly protein	PI:*NP_049802.1* Product:baseplate hub assembly protein
PI:*QXN75891.1* Product:baseplate hub subunit	PI:*NP_049803.1* Product:base plate hub subunit
PI:*QXN75892.1* Product:baseplate hub distal subunit	PI:*NP_049804.2* Product:base plate distal hub subunit

PI:*QXN75893.1*<br>Product:baseplate hub subunit tail length determinator	PI:*NP_049805.1*<br>Product:gp29 baseplate hub subunit
PI:*QXN75894.1*<br>Product:tail-tube assembly protein	PI:*NP_049806.1*<br>Product:baseplate tail-tube junction protein
PI:*QXN75895.1*<br>Product:baseplate tail-tube initiator	PI:*NP_049807.1*<br>Product:baseplate subunit
PI:*QXN75896.1*<br>Product:hypothetical protein	PI:*NP_049808.1*<br>Product:Alt.-3 conserved hypothetical protein
PI:*QXN75898.1*<br>Product:NAD(+)--arginine ADP-ribosyltransferase	PI:*NP_049811.1*<br>Product:RNA polymerase ADP-ribosylase
PI:*QXN75899.1*<br>Product:hypothetical protein	PI:*NP_049812.1*<br>Product:Alt.1 conserved hypothetical protein
PI:*QXN75900.1*<br>Product:DNA ligase	PI:*NP_049813.1*<br>Product:DNA ligase
PI:*QXN75901.1*<br>Product:hypothetical protein	PI:*NP_049814.1*<br>Product:DUF3045 domain-containing protein
PI:*QXN75902.1*<br>Product:hypothetical protein	PI:*NP_049815.1*<br>Product:hypothetical protein
PI:*QXN75903.1*<br>Product:hypothetical protein	PI:*NP_049816.1*<br>Product:hypothetical protein
PI:*QXN75904.1*<br>Product:hypothetical protein	PI:*NP_049818.1*<br>Product:gp30.4 conserved hypothetical protein
PI:*QXN75905.1*<br>Product:hypothetical protein	PI:*NP_049819.1*<br>Product:gp30.5 hypothetical protein
PI:*QXN75906.1*<br>Product:hypothetical protein	PI:*NP_049820.1*<br>Product:gp30.6 conserved hypothetical protein
PI:*QXN75907.1*<br>Product:hypothetical protein	PI:*NP_049821.1*<br>Product:gp30.7 conserved hypothetical protein
PI:*QXN75908.1*<br>Product:hypothetical protein	PI:*NP_049822.1*<br>Product:gp30.8 conserved hypothetical protein
PI:*QXN75909.1*<br>Product:hypothetical protein	PI:*NP_049823.1*<br>Product:gp30.9 conserved hypothetical protein
PI:*QXN75910.1*<br>Product:lysis inhibition accessory protein	PI:*NP_049824.1*<br>Product:rIII lysis inhibition accessory protein
PI:*QXN75911.1*<br>Product:head assembly co-chaperone	PI:*NP_049825.1*<br>Product:co-chaperone GroES family protein
PI:*QXN75912.1*<br>Product:hypothetical protein	PI:*NP_049826.1*<br>Product:SH3 beta-barrel fold-containing protein
PI:*QXN75913.1*<br>Product:hypothetical protein	PI:*NP_049827.1*<br>Product:gp31.2 hypothetical protein
PI:*QXN75914.1*<br>Product:deoxycytidylate deaminase	PI:*NP_049828.1*<br>Product:dCMP deaminase
PI:*QXN75915.1*<br>Product:hypothetical protein	PI:*NP_049829.1*<br>Product:Cd.1 hypothetical protein
PI:*QXN75916.1*<br>Product:hypothetical protein	PI:*NP_049830.1*<br>Product:Cd.2 conserved hypothetical protein

PI:*QXN75917.1*<br>Product:hypothetical protein	PI:*NP_049831.1*<br>Product:Cd.3 conserved hypothetical protein
PI:*QXN75918.1*<br>Product:hypothetical protein	PI:*NP_049832.1*<br>Product:Cd.4 conserved hypothetical protein
PI:*QXN75919.1*<br>Product:hypothetical protein	PI:*NP_049833.1*<br>Product:Cd.5 hypothetical protein
PI:*QXN75920.1*<br>Product:polynucleotide kinase	PI:*NP_049834.1*<br>Product:AAA family ATPase
PI:*QXN75922.1*<br>Product:hypothetical protein	PI:*NP_049835.1*<br>Product:PseT.1 conserved hypothetical protein
PI:*QXN75923.1*<br>Product:hypothetical protein	PI:*NP_049836.2*<br>Product:PseT.2 conserved hypothetical protein
PI:*QXN75924.1*<br>Product:hypothetical protein	PI:*NP_049837.1*<br>Product:PseT.3 conserved hypothetical predicted membrane
PI:*QXN75925.1*<br>Product:inhibitor of host transcription	PI:*NP_049838.1*<br>Product:Alc inhibitor of host transcription
PI:*QXN75926.1*<br>Product:RNA ligase	PI:*NP_049839.1*<br>Product:T4 RnlA family RNA ligase
PI:*QXN75927.1*<br>Product:endonuclease	PI:*NP_049840.1*<br>Product:GIY-YIG nuclease family protein
PI:*QXN75928.1*<br>Product:ribonucleoside-diphosphate reductase	PI:*NP_049841.1*<br>Product:ribonucleotide-diphosphate reductase subunit
PI:*QXN75929.1*<br>Product:ribonucleoside-diphosphate reductase	PI:*NP_049845.1*<br>Product:ribonucleoside-diphosphate reductase subunit
PI:*QXN75930.1*<br>Product:hypothetical protein	PI:*NP_049846.1*<br>Product:DUF4326 domain-containing protein
PI:*QXN75931.1*<br>Product:hypothetical protein	PI:*NP_049847.1*<br>Product:NrdA.2 conserved hypothetical protein
PI:*QXN75932.1*<br>Product:thymidylate synthase	PI:*NP_049848.1*<br>Product:thymidylate synthase
PI:*QXN75934.1*<br>Product:dihydrofolate reductase	PI:*NP_049850.1*<br>Product:dihydrofolate reductase
PI:*QXN75937.1*<br>Product:hypothetical protein	PI:*NP_049851.1*<br>Product:DUF5417 domain-containing protein
PI:*QXN75938.1*<br>Product:hypothetical protein	PI:*NP_049852.1*<br>Product:Frd.2 conserved hypothetical protein
PI:*QXN75939.1*<br>Product:hypothetical protein	PI:*NP_049853.1*<br>Product:Frd.3 hypothetical protein
PI:*QXN75940.1*<br>Product:helix-destabilizing protein	PI:*NP_049854.1*<br>Product:single-stranded DNA-binding protein
PI:*QXN75941.1*<br>Product:loader of DNA helicase and single-stranded	PI:*NP_049856.1*<br>Product:59 protein
PI:*QXN75942.1*<br>Product:hypothetical protein	PI:*NP_049857.1*<br>Product:late promoter transcription accessory protein
PI:*QXN75943.1*<br>Product:double-stranded DNA-binding protein	PI:*NP_049858.1*<br>Product:double-stranded DNA binding protein

PI:*QXN75944.1*<br>Product:ribonuclease H	PI:*NP_049859.1*<br>Product:RnaseH
PI:*QXN75945.1*<br>Product:long-tail fiber proximal subunit	PI:*NP_049860.1*<br>Product:tail protein
PI:*QXN75946.1*<br>Product:long-tail fiber connector	PI:*NP_049861.1*<br>Product:gp35 hinge connector of long tail fiber
PI:*QXN75947.1*<br>Product:long-tail fiber protein	PI:*NP_049862.1*<br>Product:tail fiber protein
PI:*QXN75948.1*<br>Product:long-tail fiber protein	PI:*NP_049863.1*<br>Product:gp37 long tail fiber
PI:*QXN75950.1*<br>Product:holin	PI:*NP_049865.1*<br>Product:t holin lysis mediator
PI:*QXN75951.1*<br>Product:anti-sigma factor	PI:*NP_049866.1*<br>Product:AsiA anti-sigma 70 protein
PI:*QXN75952.1*<br>Product:anti-sigma factor	PI:*NP_049867.1*<br>Product:AsiA.1 hypothetical protein
PI:*QXN75953.1*<br>Product:baseplate wedge tail fiber connector	PI:*NP_049868.1*<br>Product:Arn inhibitor of MrcBC restriction endonuclease
PI:*QXN75955.1*<br>Product:anti-restriction nuclease	PI:*NP_049870.1*<br>Product:Arn.2 conserved hypothetical protein
PI:*QXN75956.1*<br>Product:anti-restriction nuclease	PI:*NP_049871.1*<br>Product:Arn.3 conserved hypothetical protein
PI:*QXN75957.1*<br>Product:hypothetical protein	PI:*NP_049872.2*<br>Product:Arn.4 conserved hypothetical protein
PI:*QXN75958.1*<br>Product:middle transcription regulatory protein	PI:*NP_049873.1*<br>Product:flagellar motor protein MotA
PI:*QXN75959.1*<br>Product:hypothetical protein	PI:*NP_049874.1*<br>Product:MotA.1 hypothetical predicted periplasmic
PI:*QXN75960.1*<br>Product:DNA topoisomerase medium subunit	PI:*NP_049875.1*<br>Product:DNA topisomerase II medium subunit
PI:*QXN75961.1*<br>Product:acridine resistance protein	PI:*NP_049877.1*<br>Product:Ac acridine resistance protein
PI:*QXN75962.1*<br>Product:nucleoid disruption protein	PI:*NP_049879.1*<br>Product:naphthalene 1
PI:*QXN75963.1*<br>Product:hypothetical protein	PI:*NP_049880.1*<br>Product:naphthalene 1
PI:*QXN75964.1*<br>Product:outer membrane protein	PI:*NP_049881.1*<br>Product:Ndd.2 hypothetical predicted outer membrane
PI:*QXN75965.1*<br>Product:hypothetical protein	PI:*NP_049882.1*<br>Product:Ndd.2a hypothetical predicted periplasmic
PI:*QXN75966.1*<br>Product:inner membrane protein	PI:*NP_049884.1*<br>Product:Ndd.4 hypothetical predicted inner membrane
PI:*QXN75967.1*<br>Product:hypothetical protein	PI:*NP_049885.1*<br>Product:Ndd.5 hypothetical predicted outer membrane
PI:*QXN75969.1*<br>Product:endonuclease IV	PI:*NP_049887.1*<br>Product:DenB DNA endonuclease IV

PI:*QXN75970.1*<br>Product:hypothetical protein	PI:*NP_049888.1*<br>Product:DenB.1 hypothetical protein
PI:*QXN75971.1*<br>Product:lysis ihibitor rIIB	PI:*NP_049889.1*<br>Product:helix-turn-helix domain-containing protein
PI:*QXN75972.1*<br>Product:protector from prophage-induced early lysis	PI:*NP_049616.1*<br>Product:rIIA protector from prophage-induced early
PI:*QXN75973.1*<br>Product:hypothetical protein	PI:*NP_049617.1*<br>Product:rIIA.1 hypothetical protein
PI:*QXN75974.1*<br>Product:DNA topoisomerase subunit	PI:*NP_049618.1*<br>Product:gp60 topoisomerase II
PI:*QXN75976.1*<br>Product:homing endonuclease	PI:*NP_049619.1*<br>Product:gp60.1 hypothetical protein
PI:*QXN75977.1*<br>Product:DNA topoisomerase large subunit	PI:*NP_049621.1*<br>Product:DNA topoisomerase II large subunit
PI:*QXN75978.1*<br>Product:hypothetical protein	PI:*NP_049622.1*<br>Product:gp39.1 hypothetical protein
PI:*QXN75979.1*<br>Product:hypothetical protein	PI:*NP_049623.1*<br>Product:gp39.2 hypothetical protein
PI:*QXN75980.1*<br>Product:mRNA metabolism modulator	PI:*NP_049624.1*<br>Product:goF mRNA metabolism modulator
PI:*QXN75981.1*<br>Product:modifier of suppressor tRNAs	PI:*NP_049625.1*<br>Product:cytochrome C biogenesis protein CcmE
PI:*QXN75982.1*<br>Product:transcriptional regulator	PI:*NP_049626.1*<br>Product:MotB modifier of transcription
PI:*QXN75984.1*<br>Product:hypothetical protein	PI:*NP_049628.1*<br>Product:MotB.2 hypothetical protein

**Escherichia phage BF17**<br>***MW822008.1***	**Escherichia phage PBECO4**<br>***KC295538.1***
PI:*QXN75985.1*<br>Product:hypothetical protein	PI:*AGC34959.1*<br>Product:hypothetical protein
PI:*QXN75986.1*<br>Product:hypothetical protein	PI:*AGC34958.1*<br>Product:hypothetical protein
PI:*QXN75987.1*<br>Product:hypothetical protein	PI:*AGC34957.1*<br>Product:hypothetical protein
PI:*QXN75988.1*<br>Product:hypothetical protein	PI:*AGC34956.1*<br>Product:hypothetical protein
PI:*QXN75989.1*<br>Product:hypothetical protein	PI:*AGC34955.1*<br>Product:hypothetical protein
PI:*QXN75990.1*<br>Product:hypothetical protein	PI:*AGC34954.1*<br>Product:hypothetical protein
PI:*QXN75991.1*<br>Product:hypothetical protein	PI:*AGC34953.1*<br>Product:hypothetical protein
PI:*QXN75992.1*<br>Product:hypothetical protein	PI:*AGC34952.1*<br>Product:hypothetical protein
PI:*QXN75993.1*<br>Product:hypothetical protein	PI:*AGC34949.1*<br>Product:hypothetical protein

PI:*QXN75995.1*<br>Product:hypothetical protein	PI:*AGC34948.1*<br>Product:hypothetical protein
PI:*QXN75997.1*<br>Product:hypothetical protein	PI:*AGC34947.1*<br>Product:hypothetical protein
PI:*QXN75998.1*<br>Product:hypothetical protein	PI:*AGC34946.1*<br>Product:hypothetical protein
PI:*QXN76000.1*<br>Product:hypothetical protein	PI:*AGC34945.1*<br>Product:hypothetical protein
PI:*QXN76001.1*<br>Product:hypothetical protein	PI:*AGC34944.1*<br>Product:hypothetical protein
PI:*QXN76002.1*<br>Product:hypothetical protein	PI:*AGC34943.1*<br>Product:hypothetical protein
PI:*QXN76003.1*<br>Product:hypothetical protein	PI:*AGC34942.1*<br>Product:hypothetical protein
PI:*QXN76004.1*<br>Product:hypothetical protein	PI:*AGC34941.1*<br>Product:hypothetical protein
PI:*QXN76006.1*<br>Product:hypothetical protein	PI:*AGC34939.1*<br>Product:hypothetical protein
PI:*QXN76007.1*<br>Product:hypothetical protein	PI:*AGC34938.1*<br>Product:hypothetical protein
PI:*QXN76008.1*<br>Product:hypothetical protein	PI:*AGC34937.1*<br>Product:hypothetical protein
PI:*QXN76010.1*<br>Product:hypothetical protein	PI:*AGC34936.1*<br>Product:hypothetical protein
PI:*QXN76011.1*<br>Product:hypothetical protein	PI:*AGC34935.1*<br>Product:hypothetical protein
PI:*QXN76012.1*<br>Product:hypothetical protein	PI:*AGC34934.1*<br>Product:hypothetical protein
PI:*QXN76015.1*<br>Product:hypothetical protein	PI:*AGC34931.1*<br>Product:hypothetical protein
PI:*QXN76016.1*<br>Product:hypothetical protein	PI:*AGC34930.1*<br>Product:hypothetical protein
PI:*QXN76017.1*<br>Product:hypothetical protein	PI:*AGC34929.1*<br>Product:hypothetical protein
PI:*QXN76018.1*<br>Product:hypothetical protein	PI:*AGC34928.1*<br>Product:hypothetical protein
PI:*QXN76019.1*<br>Product:hypothetical protein	PI:*AGC34926.1*<br>Product:hypothetical protein
PI:*QXN76020.1*<br>Product:hypothetical protein	PI:*AGC34925.1*<br>Product:hypothetical protein
PI:*QXN76021.1*<br>Product:hypothetical protein	PI:*AGC34924.1*<br>Product:hypothetical protein
PI:*QXN76022.1*<br>Product:hypothetical protein	PI:*AGC34923.1*<br>Product:hypothetical protein
PI:*QXN76025.1*<br>Product:hypothetical protein	PI:*AGC34922.1*<br>Product:hypothetical protein
PI:*QXN76026.1*<br>Product:hypothetical protein	PI:*AGC34921.1*<br>Product:hypothetical protein

PI:*QXN76027.1* Product:hypothetical protein	PI:*AGC34920.1* Product:hypothetical protein
PI:*QXN76028.1* Product:hypothetical protein	PI:*AGC34919.1* Product:hypothetical protein
PI:*QXN76029.1* Product:hypothetical protein	PI:*AGC34918.1* Product:hypothetical protein
PI:*QXN76030.1* Product:hypothetical protein	PI:*AGC34917.1* Product:hypothetical protein
PI:*QXN76031.1* Product:hypothetical protein	PI:*AGC34916.1* Product:hypothetical protein
PI:*QXN76033.1* Product:hypothetical protein	PI:*AGC34915.1* Product:hypothetical protein
PI:*QXN76034.1* Product:hypothetical protein	PI:*AGC34914.1* Product:hypothetical protein
PI:*QXN76036.1* Product:hypothetical protein	PI:*AGC34912.1* Product:hypothetical protein
PI:*QXN76037.1* Product:hypothetical protein	PI:*AGC34911.1* Product:hypothetical protein
PI:*QXN76038.1* Product:hypothetical protein	PI:*AGC34910.1* Product:hypothetical protein
PI:*QXN76039.1* Product:hypothetical protein	PI:*AGC34909.1* Product:hypothetical protein
PI:*QXN76040.1* Product:hypothetical protein	PI:*AGC34908.1* Product:hypothetical protein
PI:*QXN76042.1* Product:hypothetical protein	PI:*AGC34907.1* Product:hypothetical protein
PI:*QXN76044.1* Product:hypothetical protein	PI:*AGC34906.1* Product:hypothetical protein
PI:*QXN76045.1* Product:hypothetical protein	PI:*AGC34904.1* Product:hypothetical protein
PI:*QXN76046.1* Product:hypothetical protein	PI:*AGC34903.1* Product:hypothetical protein
PI:*QXN76048.1* Product:hypothetical protein	PI:*AGC34902.1* Product:hypothetical protein
PI:*QXN76050.1* Product:hypothetical protein	PI:*AGC34901.1* Product:hypothetical protein
PI:*QXN76051.1* Product:hypothetical protein	PI:*AGC34900.1* Product:hypothetical protein
PI:*QXN76054.1* Product:hypothetical protein	PI:*AGC34899.1* Product:hypothetical protein
PI:*QXN76055.1* Product:hypothetical protein	PI:*AGC34898.1* Product:hypothetical protein
PI:*QXN76057.1* Product:hypothetical protein	PI:*AGC34897.1* Product:hypothetical protein
PI:*QXN76058.1* Product:hypothetical protein	PI:*AGC34896.1* Product:hypothetical protein
PI:*QXN76061.1* Product:hypothetical protein	PI:*AGC34895.1* Product:hypothetical protein

PI:*QXN76062.1* Product:hypothetical protein	PI:*AGC34894.1* Product:hypothetical protein
PI:*QXN76063.1* Product:hypothetical protein	PI:*AGC34893.1* Product:hypothetical protein
PI:*QXN76064.1* Product:hypothetical protein	PI:*AGC34892.1* Product:hypothetical protein
PI:*QXN76066.1* Product:hypothetical protein	PI:*AGC34891.1* Product:hypothetical protein
PI:*QXN76067.1* Product:hypothetical protein	PI:*AGC34890.1* Product:hypothetical protein
PI:*QXN76068.1* Product:hypothetical protein	PI:*AGC34889.1* Product:hypothetical protein
PI:*QXN76071.1* Product:hypothetical protein	PI:*AGC34888.1* Product:hypothetical protein
PI:*QXN76073.1* Product:hypothetical protein	PI:*AGC34887.1* Product:hypothetical protein
PI:*QXN76074.1* Product:hypothetical protein	PI:*AGC34886.1* Product:hypothetical protein
PI:*QXN76075.1* Product:hypothetical protein	PI:*AGC34885.1* Product:hypothetical protein
PI:*QXN76076.1* Product:hypothetical protein	PI:*AGC34884.1* Product:hypothetical protein
PI:*QXN76077.1* Product:hypothetical protein	PI:*AGC34883.1* Product:hypothetical protein
PI:*QXN76078.1* Product:hypothetical protein	PI:*AGC34882.1* Product:hypothetical protein
PI:*QXN76079.1* Product:hypothetical protein	PI:*AGC34881.1* Product:hypothetical protein
PI:*QXN76080.1* Product:hypothetical protein	PI:*AGC34880.1* Product:hypothetical protein
PI:*QXN76081.1* Product:hypothetical protein	PI:*AGC34879.1* Product:hypothetical protein
PI:*QXN76082.1* Product:hypothetical protein	PI:*AGC34878.1* Product:hypothetical protein
PI:*QXN76083.1* Product:hypothetical protein	PI:*AGC34877.1* Product:hypothetical protein
PI:*QXN76086.1* Product:hypothetical protein	PI:*AGC34875.1* Product:hypothetical protein
PI:*QXN76089.1* Product:hypothetical protein	PI:*AGC34874.1* Product:hypothetical protein
PI:*QXN76090.1* Product:hypothetical protein	PI:*AGC34873.1* Product:hypothetical protein
PI:*QXN76091.1* Product:hypothetical protein	PI:*AGC34872.1* Product:hypothetical protein
PI:*QXN76092.1* Product:hypothetical protein	PI:*AGC34871.1* Product:hypothetical protein
PI:*QXN76093.1* Product:hypothetical protein	PI:*AGC34870.1* Product:hypothetical protein

PI:*QXN76094.1*<br>Product:hypothetical protein	PI:*AGC34869.1*<br>Product:hypothetical protein
PI:*QXN76095.1*<br>Product:hypothetical protein	PI:*AGC34868.1*<br>Product:hypothetical protein
PI:*QXN76096.1*<br>Product:hypothetical protein	PI:*AGC34867.1*<br>Product:hypothetical protein
PI:*QXN76097.1*<br>Product:hypothetical protein	PI:*AGC34866.1*<br>Product:hypothetical protein
PI:*QXN76098.1*<br>Product:hypothetical protein	PI:*AGC34865.1*<br>Product:hypothetical protein
PI:*QXN76099.1*<br>Product:hypothetical protein	PI:*AGC34864.1*<br>Product:hypothetical protein
PI:*QXN76101.1*<br>Product:hypothetical protein	PI:*AGC34863.1*<br>Product:hypothetical protein
PI:*QXN76102.1*<br>Product:hypothetical protein	PI:*AGC34862.1*<br>Product:hypothetical protein
PI:*QXN76103.1*<br>Product:hypothetical protein	PI:*AGC34861.1*<br>Product:hypothetical protein
PI:*QXN76104.1*<br>Product:hypothetical protein	PI:*AGC34860.1*<br>Product:hypothetical protein
PI:*QXN76105.1*<br>Product:hypothetical protein	PI:*AGC34859.1*<br>Product:hypothetical protein
PI:*QXN76106.1*<br>Product:hypothetical protein	PI:*AGC34858.1*<br>Product:hypothetical protein
PI:*QXN76107.1*<br>Product:hypothetical protein	PI:*AGC34857.1*<br>Product:hypothetical protein
PI:*QXN76108.1*<br>Product:hypothetical protein	PI:*AGC34856.1*<br>Product:hypothetical protein
PI:*QXN76109.1*<br>Product:hypothetical protein	PI:*AGC34855.1*<br>Product:hypothetical protein
PI:*QXN76110.1*<br>Product:hypothetical protein	PI:*AGC34854.1*<br>Product:hypothetical protein
PI:*QXN76111.1*<br>Product:hypothetical protein	PI:*AGC34853.1*<br>Product:hypothetical protein
PI:*QXN76112.1*<br>Product:hypothetical protein	PI:*AGC34852.1*<br>Product:hypothetical protein
PI:*QXN76113.1*<br>Product:hypothetical protein	PI:*AGC34851.1*<br>Product:hypothetical protein
PI:*QXN76114.1*<br>Product:hypothetical protein	PI:*AGC34850.1*<br>Product:hypothetical protein
PI:*QXN76115.1*<br>Product:hypothetical protein	PI:*AGC34849.1*<br>Product:hypothetical protein
PI:*QXN76116.1*<br>Product:hypothetical protein	PI:*AGC34848.1*<br>Product:hypothetical protein
PI:*QXN76117.1*<br>Product:hypothetical protein	PI:*AGC34847.1*<br>Product:hypothetical protein
PI:*QXN76118.1*<br>Product:hypothetical protein	PI:*AGC34846.1*<br>Product:hypothetical protein

PI:*QXN76119.1*<br>Product:hypothetical protein	PI:*AGC34845.1*<br>Product:hypothetical protein
PI:*QXN76120.1*<br>Product:hypothetical protein	PI:*AGC34844.1*<br>Product:hypothetical protein
PI:*QXN76121.1*<br>Product:hypothetical protein	PI:*AGC34843.1*<br>Product:hypothetical protein
PI:*QXN76122.1*<br>Product:hypothetical protein	PI:*AGC34842.1*<br>Product:hypothetical protein
PI:*QXN76123.1*<br>Product:hypothetical protein	PI:*AGC34841.1*<br>Product:hypothetical protein
PI:*QXN76124.1*<br>Product:hypothetical protein	PI:*AGC34840.1*<br>Product:hypothetical protein
PI:*QXN76125.1*<br>Product:hypothetical protein	PI:*AGC34839.1*<br>Product:hypothetical protein
PI:*QXN76126.1*<br>Product:hypothetical protein	PI:*AGC34838.1*<br>Product:hypothetical protein
PI:*QXN76127.1*<br>Product:hypothetical protein	PI:*AGC34837.1*<br>Product:hypothetical protein
PI:*QXN76128.1*<br>Product:hypothetical protein	PI:*AGC34836.1*<br>Product:hypothetical protein
PI:*QXN76129.1*<br>Product:hypothetical protein	PI:*AGC34835.1*<br>Product:hypothetical protein
PI:*QXN76131.1*<br>Product:hypothetical protein	PI:*AGC34834.1*<br>Product:hypothetical protein
PI:*QXN76132.1*<br>Product:hypothetical protein	PI:*AGC34833.1*<br>Product:hypothetical protein
PI:*QXN76133.1*<br>Product:hypothetical protein	PI:*AGC34832.1*<br>Product:hypothetical protein
PI:*QXN76134.1*<br>Product:hypothetical protein	PI:*AGC34831.1*<br>Product:hypothetical protein
PI:*QXN76135.1*<br>Product:hypothetical protein	PI:*AGC34830.1*<br>Product:hypothetical protein
PI:*QXN76136.1*<br>Product:hypothetical protein	PI:*AGC34829.1*<br>Product:hypothetical protein
PI:*QXN76137.1*<br>Product:methylase	PI:*AGC34828.1*<br>Product:methylase
PI:*QXN76138.1*<br>Product:hypothetical protein	PI:*AGC34827.1*<br>Product:putative cytitidyltransferase
PI:*QXN76139.1*<br>Product:hypothetical protein	PI:*AGC34826.1*<br>Product:hypothetical protein
PI:*QXN76140.1*<br>Product:hypothetical protein	PI:*AGC34825.1*<br>Product:hypothetical protein
PI:*QXN76141.1*<br>Product:hypothetical protein	PI:*AGC34824.1*<br>Product:hypothetical protein
PI:*QXN76142.1*<br>Product:hypothetical protein	PI:*AGC34823.1*<br>Product:hypothetical protein
PI:*QXN76143.1*<br>Product:hypothetical protein	PI:*AGC34822.1*<br>Product:hypothetical protein

PI:*QXN76144.1*<br>Product:hypothetical protein	PI:*AGC34821.1*<br>Product:hypothetical protein
PI:*QXN76145.1*<br>Product:hypothetical protein	PI:*AGC34820.1*<br>Product:hypothetical protein
PI:*QXN76147.1*<br>Product:hypothetical protein	PI:*AGC34818.1*<br>Product:hypothetical protein
PI:*QXN76148.1*<br>Product:hypothetical protein	PI:*AGC34817.1*<br>Product:hypothetical protein
PI:*QXN76150.1*<br>Product:hypothetical protein	PI:*AGC34816.1*<br>Product:hypothetical protein
PI:*QXN76151.1*<br>Product:hypothetical protein	PI:*AGC34815.1*<br>Product:hypothetical protein
PI:*QXN76152.1*<br>Product:hypothetical protein	PI:*AGC34814.1*<br>Product:hypothetical protein
PI:*QXN76153.1*<br>Product:hypothetical protein	PI:*AGC34813.1*<br>Product:hypothetical protein
PI:*QXN76154.1*<br>Product:hypothetical protein	PI:*AGC34812.1*<br>Product:hypothetical protein
PI:*QXN76155.1*<br>Product:hypothetical protein	PI:*AGC34811.1*<br>Product:hypothetical protein
PI:*QXN76156.1*<br>Product:hypothetical protein	PI:*AGC34810.1*<br>Product:hypothetical protein
PI:*QXN76157.1*<br>Product:RNA ligase	PI:*AGC34809.1*<br>Product:RNA ligase
PI:*QXN76158.1*<br>Product:hypothetical protein	PI:*AGC34808.1*<br>Product:hypothetical protein
PI:*QXN76159.1*<br>Product:hypothetical protein	PI:*AGC34807.1*<br>Product:hypothetical protein
PI:*QXN76160.1*<br>Product:hypothetical protein	PI:*AGC34806.1*<br>Product:hypothetical protein
PI:*QXN76161.1*<br>Product:polynucleotide kinase	PI:*AGC34805.1*<br>Product:polynucleotide 5'-kinase and 3'-phosphatase
PI:*QXN76162.1*<br>Product:hypothetical protein	PI:*AGC34803.1*<br>Product:hypothetical protein
PI:*QXN76163.1*<br>Product:hypothetical protein	PI:*AGC34802.1*<br>Product:hypothetical protein
PI:*QXN76164.1*<br>Product:hypothetical protein	PI:*AGC34801.1*<br>Product:hypothetical protein
PI:*QXN76165.1*<br>Product:hypothetical protein	PI:*AGC34800.1*<br>Product:hypothetical protein
PI:*QXN76166.1*<br>Product:hypothetical protein	PI:*AGC34799.1*<br>Product:hypothetical protein
PI:*QXN76167.1*<br>Product:hypothetical protein	PI:*AGC34798.1*<br>Product:hypothetical protein
PI:*QXN76168.1*<br>Product:hypothetical protein	PI:*AGC34797.1*<br>Product:hypothetical protein
PI:*QXN76169.1*<br>Product:hypothetical protein	PI:*AGC34796.1*<br>Product:hypothetical protein

PI:*QXN76171.1*<br>Product:hypothetical protein	PI:*AGC34795.1*<br>Product:hypothetical protein
PI:*QXN76172.1*<br>Product:serine/threonine-protein phosphatase	PI:*AGC34794.1*<br>Product:putative serine/threonine protein phosphatase
PI:*QXN76173.1*<br>Product:hypothetical protein	PI:*AGC34793.1*<br>Product:hypothetical protein
PI:*QXN76174.1*<br>Product:hypothetical protein	PI:*AGC34792.1*<br>Product:hypothetical protein
PI:*QXN76175.1*<br>Product:hypothetical protein	PI:*AGC34791.1*<br>Product:hypothetical protein
PI:*QXN76176.1*<br>Product:hypothetical protein	PI:*AGC34790.1*<br>Product:hypothetical protein
PI:*QXN76177.1*<br>Product:DNA ligase	PI:*AGC34789.1*<br>Product:DNA ligase
PI:*QXN76178.1*<br>Product:serine/threonine-protein phosphatase	PI:*AGC34788.1*<br>Product:Ser/Thr protein phosphatase family protein
PI:*QXN76179.1*<br>Product:hypothetical protein	PI:*AGC34787.1*<br>Product:hypothetical protein
PI:*QXN76180.1*<br>Product:hypothetical protein	PI:*AGC34786.1*<br>Product:hypothetical protein
PI:*QXN76181.1*<br>Product:protease	PI:*AGC34785.1*<br>Product:putative ATP-dependent Clp protease proteolytic
PI:*QXN76182.1*<br>Product:hypothetical protein	PI:*AGC34784.1*<br>Product:hypothetical protein
PI:*QXN76183.1*<br>Product:hypothetical protein	PI:*AGC34783.1*<br>Product:hypothetical protein
PI:*QXN76184.1*<br>Product:hypothetical protein	PI:*AGC34782.1*<br>Product:hypothetical protein
PI:*QXN76185.1*<br>Product:hypothetical protein	PI:*AGC34781.1*<br>Product:hypothetical protein
PI:*QXN76186.1*<br>Product:deoxycytidylate deaminase	PI:*AGC34779.1*<br>Product:dCMP deaminase
PI:*QXN76188.1*<br>Product:structural protein	PI:*AGC34778.1*<br>Product:putative structural protein
PI:*QXN76189.1*<br>Product:hypothetical protein	PI:*AGC34777.1*<br>Product:hypothetical protein
PI:*QXN76191.1*<br>Product:hypothetical protein	PI:*AGC34776.1*<br>Product:hypothetical protein
PI:*QXN76193.1*<br>Product:NAD-dependent protein deacylase	PI:*AGC34775.1*<br>Product:putative Sir2-like protein
PI:*QXN76194.1*<br>Product:nudix hydrolase	PI:*AGC34774.1*<br>Product:nudix hydrolase
PI:*QXN76195.1*<br>Product:hypothetical protein	PI:*AGC34773.1*<br>Product:hypothetical protein
PI:*QXN76196.1*<br>Product:hypothetical protein	PI:*AGC34772.1*<br>Product:hypothetical protein

PI:*QXN76197.1*<br>Product:hypothetical protein	PI:*AGC34771.1*<br>Product:hypothetical protein
PI:*QXN76198.1*<br>Product:hypothetical protein	PI:*AGC34770.1*<br>Product:hypothetical protein
PI:*QXN76199.1*<br>Product:hypothetical protein	PI:*AGC34769.1*<br>Product:hypothetical protein
PI:*QXN76200.1*<br>Product:hypothetical protein	PI:*AGC34768.1*<br>Product:hypothetical protein
PI:*QXN76201.1*<br>Product:hypothetical protein	PI:*AGC34767.1*<br>Product:hypothetical protein
PI:*QXN76203.1*<br>Product:WGR domain-containing protein	PI:*AGC34766.1*<br>Product:WGR domain-containing protein
PI:*QXN76204.1*<br>Product:hypothetical protein	PI:*AGC34765.1*<br>Product:hypothetical protein
PI:*QXN76205.1*<br>Product:hypothetical protein	PI:*AGC34764.1*<br>Product:hypothetical protein
PI:*QXN76206.1*<br>Product:hypothetical protein	PI:*AGC34763.1*<br>Product:hypothetical protein
PI:*QXN76207.1*<br>Product:hypothetical protein	PI:*AGC34762.1*<br>Product:hypothetical protein
PI:*QXN76208.1*<br>Product:hypothetical protein	PI:*AGC34761.1*<br>Product:hypothetical protein
PI:*QXN76209.1*<br>Product:hypothetical protein	PI:*AGC34760.1*<br>Product:hypothetical protein
PI:*QXN76211.1*<br>Product:hypothetical protein	PI:*AGC34759.1*<br>Product:hypothetical protein
PI:*QXN76212.1*<br>Product:hypothetical protein	PI:*AGC34758.1*<br>Product:hypothetical protein
PI:*QXN76213.1*<br>Product:hypothetical protein	PI:*AGC34757.1*<br>Product:hypothetical protein
PI:*QXN76215.1*<br>Product:anaerobic ribonucleoside-triphosphate reductase	PI:*AGC34756.1*<br>Product:anaerobic NTP reductase large subunit
PI:*QXN76216.1*<br>Product:hypothetical protein	PI:*AGC34755.1*<br>Product:hypothetical protein
PI:*QXN76217.1*<br>Product:anaerobic ribonucleoside-triphosphate reductase	PI:*AGC34754.1*<br>Product:anaerobic nucleotide reductase subunit
PI:*QXN76218.1*<br>Product:hypothetical protein	PI:*AGC34753.1*<br>Product:hypothetical protein
PI:*QXN76219.1*<br>Product:hypothetical protein	PI:*AGC34752.1*<br>Product:hypothetical protein
PI:*QXN76220.1*<br>Product:hypothetical protein	PI:*AGC34751.1*<br>Product:hypothetical protein
PI:*QXN76221.1*<br>Product:hypothetical protein	PI:*AGC34750.1*<br>Product:hypothetical protein
PI:*QXN76222.1*<br>Product:hypothetical protein	PI:*AGC34749.1*<br>Product:hypothetical protein

PI:*QXN76223.1*<br>Product:hypothetical protein	PI:*AGC34748.1*<br>Product:hypothetical protein
PI:*QXN76224.1*<br>Product:hypothetical protein	PI:*AGC34747.1*<br>Product:hypothetical protein
PI:*QXN76225.1*<br>Product:hypothetical protein	PI:*AGC34746.1*<br>Product:hypothetical protein
PI:*QXN76226.1*<br>Product:hypothetical protein	PI:*AGC34745.1*<br>Product:hypothetical protein
PI:*QXN76227.1*<br>Product:hypothetical protein	PI:*AGC34744.1*<br>Product:hypothetical protein
PI:*QXN76228.1*<br>Product:hypothetical protein	PI:*AGC34743.1*<br>Product:hypothetical protein
PI:*QXN76229.1*<br>Product:hypothetical protein	PI:*AGC34742.1*<br>Product:hypothetical protein
PI:*QXN76230.1*<br>Product:hypothetical protein	PI:*AGC34741.1*<br>Product:hypothetical protein
PI:*QXN76231.1*<br>Product:hypothetical protein	PI:*AGC34740.1*<br>Product:hypothetical protein
PI:*QXN76232.1*<br>Product:hypothetical protein	PI:*AGC34739.1*<br>Product:hypothetical protein
PI:*QXN76233.1*<br>Product:hypothetical protein	PI:*AGC34738.1*<br>Product:hypothetical protein
PI:*QXN76234.1*<br>Product:hypothetical protein	PI:*AGC34737.1*<br>Product:hypothetical protein
PI:*QXN76235.1*<br>Product:hypothetical protein	PI:*AGC34736.1*<br>Product:hypothetical protein
PI:*QXN76236.1*<br>Product:hypothetical protein	PI:*AGC34735.1*<br>Product:hypothetical protein
PI:*QXN76237.1*<br>Product:hypothetical protein	PI:*AGC34734.1*<br>Product:hypothetical protein
PI:*QXN76238.1*<br>Product:hypothetical protein	PI:*AGC34733.1*<br>Product:hypothetical protein
PI:*QXN76239.1*<br>Product:hypothetical protein	PI:*AGC34732.1*<br>Product:hypothetical protein
PI:*QXN76240.1*<br>Product:hypothetical protein	PI:*AGC34731.1*<br>Product:hypothetical protein
PI:*QXN76241.1*<br>Product:hypothetical protein	PI:*AGC34730.1*<br>Product:hypothetical protein
PI:*QXN76242.1*<br>Product:hypothetical protein	PI:*AGC34729.1*<br>Product:hypothetical protein
PI:*QXN76243.1*<br>Product:hypothetical protein	PI:*AGC34728.1*<br>Product:hypothetical protein
PI:*QXN76245.1*<br>Product:hypothetical protein	PI:*AGC34727.1*<br>Product:hypothetical protein
PI:*QXN76246.1*<br>Product:hypothetical protein	PI:*AGC34726.1*<br>Product:hypothetical protein
PI:*QXN76247.1*<br>Product:hypothetical protein	PI:*AGC34725.1*<br>Product:hypothetical protein

PI:*QXN76248.1*<br>Product:hypothetical protein	PI:*AGC34724.1*<br>Product:hypothetical protein
PI:*QXN76249.1*<br>Product:hypothetical protein	PI:*AGC34723.1*<br>Product:hypothetical protein
PI:*QXN76250.1*<br>Product:hypothetical protein	PI:*AGC34722.1*<br>Product:hypothetical protein
PI:*QXN76251.1*<br>Product:hypothetical protein	PI:*AGC34721.1*<br>Product:hypothetical protein
PI:*QXN76252.1*<br>Product:lysozyme	PI:*AGC34720.1*<br>Product:putative lysozyme
PI:*QXN76253.1*<br>Product:hypothetical protein	PI:*AGC34719.1*<br>Product:hypothetical protein
PI:*QXN76254.1*<br>Product:hypothetical protein	PI:*AGC34718.1*<br>Product:hypothetical protein
PI:*QXN76255.1*<br>Product:hypothetical protein	PI:*AGC34717.1*<br>Product:hypothetical protein
PI:*QXN76256.1*<br>Product:hypothetical protein	PI:*AGC34716.1*<br>Product:hypothetical protein
PI:*QXN76257.1*<br>Product:hypothetical protein	PI:*AGC34715.1*<br>Product:hypothetical protein
PI:*QXN76258.1*<br>Product:hypothetical protein	PI:*AGC34714.1*<br>Product:hypothetical protein
PI:*QXN76259.1*<br>Product:hypothetical protein	PI:*AGC34713.1*<br>Product:hypothetical protein
PI:*QXN76260.1*<br>Product:nudix hydrolase	PI:*AGC34712.1*<br>Product:nudix hydrolase
PI:*QXN76261.1*<br>Product:hypothetical protein	PI:*AGC34711.1*<br>Product:hypothetical protein
PI:*QXN76262.1*<br>Product:hypothetical protein	PI:*AGC34710.1*<br>Product:hypothetical protein
PI:*QXN76263.1*<br>Product:hypothetical protein	PI:*AGC34709.1*<br>Product:hypothetical protein
PI:*QXN76264.1*<br>Product:hypothetical protein	PI:*AGC34708.1*<br>Product:hypothetical protein
PI:*QXN76265.1*<br>Product:hypothetical protein	PI:*AGC34707.1*<br>Product:hypothetical protein
PI:*QXN76266.1*<br>Product:hypothetical protein	PI:*AGC34706.1*<br>Product:hypothetical protein
PI:*QXN76267.1*<br>Product:hypothetical protein	PI:*AGC34705.1*<br>Product:hypothetical protein
PI:*QXN76268.1*<br>Product:hypothetical protein	PI:*AGC34704.1*<br>Product:hypothetical protein
PI:*QXN76269.1*<br>Product:deoxynucleoside monophosphate kinase	PI:*AGC34703.1*<br>Product:putative deoxynucleoside monophosphate kinase
PI:*QXN76270.1*<br>Product:tail sheath protein	PI:*AGC34702.1*<br>Product:tail sheath protein

PI:*QXN76272.1*<br>Product:hypothetical protein	PI:*AGC34701.1*<br>Product:hypothetical protein
PI:*QXN76273.1*<br>Product:hypothetical protein	PI:*AGC34700.1*<br>Product:hypothetical protein
PI:*QXN76274.1*<br>Product:hypothetical protein	PI:*AGC34699.1*<br>Product:hypothetical protein
PI:*QXN76275.1*<br>Product:head completion protein	PI:*AGC34698.1*<br>Product:head completion protein
PI:*QXN76276.1*<br>Product:hypothetical protein	PI:*AGC34697.1*<br>Product:hypothetical protein
PI:*QXN76277.1*<br>Product:hypothetical protein	PI:*AGC34696.1*<br>Product:hypothetical protein
PI:*QXN76278.1*<br>Product:hypothetical protein	PI:*AGC34695.1*<br>Product:hypothetical protein
PI:*QXN76279.1*<br>Product:hypothetical protein	PI:*AGC34694.1*<br>Product:hypothetical protein
PI:*QXN76280.1*<br>Product:hypothetical protein	PI:*AGC34693.1*<br>Product:hypothetical protein
PI:*QXN76281.1*<br>Product:hypothetical protein	PI:*AGC34692.1*<br>Product:hypothetical protein
PI:*QXN76282.1*<br>Product:hypothetical protein	PI:*AGC34691.1*<br>Product:hypothetical protein
PI:*QXN76283.1*<br>Product:ATPase	PI:*AGC34690.1*<br>Product:ATPase
PI:*QXN76284.1*<br>Product:HNH endonuclease	PI:*AGC34689.1*<br>Product:putative HNH endonuclease
PI:*QXN76285.1*<br>Product:hypothetical protein	PI:*AGC34688.1*<br>Product:hypothetical protein
PI:*QXN76286.1*<br>Product:hypothetical protein	PI:*AGC34687.1*<br>Product:hypothetical protein
PI:*QXN76287.1*<br>Product:hypothetical protein	PI:*AGC34686.1*<br>Product:hypothetical protein
PI:*QXN76288.1*<br>Product:thymidylate synthase	PI:*AGC34685.1*<br>Product:dTMP thymidylate synthase
PI:*QXN76289.1*<br>Product:hypothetical protein	PI:*AGC34684.1*<br>Product:hypothetical protein
PI:*QXN76290.1*<br>Product:hypothetical protein	PI:*AGC34682.1*<br>Product:hypothetical protein
PI:*QXN76291.1*<br>Product:long tail fiber proximal subunit	PI:*AGC34681.1*<br>Product:YadA domain protein
PI:*QXN76292.1*<br>Product:long tail fiber proximal subunit	PI:*AGC34681.1*<br>Product:YadA domain protein
PI:*QXN76293.1*<br>Product:hypothetical protein	PI:*AGC35228.1*<br>Product:hypothetical protein
PI:*QXN76294.1*<br>Product:colanic acid biosynthesis protein	PI:*AGC35227.1*<br>Product:putative colanic acid degrading protein
PI:*QXN76295.1*<br>Product:hypothetical protein	PI:*AGC35226.1*<br>Product:hypothetical protein

PI:*QXN76296.1*<br>Product:hypothetical protein	PI:*AGC35225.1*<br>Product:hypothetical protein
PI:*QXN76297.1*<br>Product:hypothetical protein	PI:*AGC35224.1*<br>Product:hypothetical protein
PI:*QXN76298.1*<br>Product:hypothetical protein	PI:*AGC35223.1*<br>Product:hypothetical protein
PI:*QXN76299.1*<br>Product:nudix hydrolase	PI:*AGC35222.1*<br>Product:putative MutT/NUDIX hydrolase family protein
PI:*QXN76300.1*<br>Product:hypothetical protein	PI:*AGC35221.1*<br>Product:hypothetical protein
PI:*QXN76301.1*<br>Product:hypothetical protein	PI:*AGC35220.1*<br>Product:hypothetical protein
PI:*QXN76302.1*<br>Product:hypothetical protein	PI:*AGC35219.1*<br>Product:hypothetical protein
PI:*QXN76303.1*<br>Product:hypothetical protein	PI:*AGC35218.1*<br>Product:hypothetical protein
PI:*QXN76304.1*<br>Product:hypothetical protein	PI:*AGC35217.1*<br>Product:hypothetical protein
PI:*QXN76305.1*<br>Product:hypothetical protein	PI:*AGC35216.1*<br>Product:hypothetical protein
PI:*QXN76306.1*<br>Product:baseplate hub subunit and tail lysozyme	PI:*AGC35215.1*<br>Product:T4-like lyzozyme
PI:*QXN76307.1*<br>Product:virion protein and lysozyme murein	PI:*AGC35214.1*<br>Product:putative virion protein and lysozyme murein
PI:*QXN76308.1*<br>Product:hypothetical protein	PI:*AGC35213.1*<br>Product:hypothetical protein
PI:*QXN76309.1*<br>Product:hypothetical protein	PI:*AGC35212.1*<br>Product:hypothetical protein
PI:*QXN76310.1*<br>Product:hypothetical protein	PI:*AGC35211.1*<br>Product:hypothetical protein
PI:*QXN76311.1*<br>Product:RNA polymerase sigma factor	PI:*AGC35210.1*<br>Product:RNA polymerase sigma factor
PI:*QXN76312.1*<br>Product:nuclease subunit	PI:*AGC35209.1*<br>Product:endonuclease subunit
PI:*QXN76313.1*<br>Product:recombination nuclease subunit	PI:*AGC35208.1*<br>Product:recombination endonuclease subunit
PI:*QXN76314.1*<br>Product:DNA endonuclease VII	PI:*AGC35207.1*<br>Product:recombination endonuclease VII
PI:*QXN76315.1*<br>Product:hypothetical protein	PI:*AGC35206.1*<br>Product:hypothetical protein
PI:*QXN76316.1*<br>Product:baseplate hub subunit	PI:*AGC35205.1*<br>Product:baseplate hub subunit
PI:*QXN76317.1*<br>Product:hypothetical protein	PI:*AGC35204.1*<br>Product:hypothetical protein
PI:*QXN76318.1*<br>Product:portal protein	PI:*AGC35203.1*<br>Product:portal vertex protein

PI:*QXN76319.1*<br>Product:hypothetical protein	PI:*AGC35202.1*<br>Product:hypothetical protein
PI:*QXN76320.1*<br>Product:hypothetical protein	PI:*AGC35201.1*<br>Product:hypothetical protein
PI:*QXN76321.1*<br>Product:prohead assembly protein	PI:*AGC35200.1*<br>Product:prohead core scaffolding protein and protease
PI:*QXN76322.1*<br>Product:hypothetical protein	PI:*AGC35199.1*<br>Product:hypothetical protein
PI:*QXN76323.1*<br>Product:major capsid protein	PI:*AGC35198.1*<br>Product:major capsid protein
PI:*QXN76324.1*<br>Product:hypothetical protein	PI:*AGC34986.1*<br>Product:hypothetical protein
PI:*QXN76325.1*<br>Product:hypothetical protein	PI:*AGC35197.1*<br>Product:hypothetical protein
PI:*QXN76326.1*<br>Product:hypothetical protein	PI:*AGC35196.1*<br>Product:hypothetical protein
PI:*QXN76327.1*<br>Product:hypothetical protein	PI:*AGC35195.1*<br>Product:hypothetical protein
PI:*QXN76328.1*<br>Product:tail fiber assembly protein	PI:*AGC35194.1*<br>Product:hypothetical protein
PI:*QXN76329.1*<br>Product:hypothetical protein	PI:*AGC35193.1*<br>Product:hypothetical protein
PI:*QXN76330.1*<br>Product:DNA polymerase	PI:*AGC35192.1*<br>Product:DNA polymerase
PI:*QXN76331.1*<br>Product:hypothetical protein	PI:*AGC35191.1*<br>Product:hypothetical protein
PI:*QXN76332.1*<br>Product:hypothetical protein	PI:*AGC35190.1*<br>Product:hypothetical protein
PI:*QXN76333.1*<br>Product:hypothetical protein	PI:*AGC35189.1*<br>Product:hypothetical protein
PI:*QXN76334.1*<br>Product:hypothetical protein	PI:*AGC35188.1*<br>Product:hypothetical protein
PI:*QXN76335.1*<br>Product:hypothetical protein	PI:*AGC35187.1*<br>Product:hypothetical protein
PI:*QXN76336.1*<br>Product:hypothetical protein	PI:*AGC35186.1*<br>Product:hypothetical protein
PI:*QXN76337.1*<br>Product:hypothetical protein	PI:*AGC35185.1*<br>Product:hypothetical protein
PI:*QXN76338.1*<br>Product:hypothetical protein	PI:*AGC35184.1*<br>Product:hypothetical protein
PI:*QXN76339.1*<br>Product:RNase H-like 5'-3' exonuclease	PI:*AGC35183.1*<br>Product:RnaseH
PI:*QXN76340.1*<br>Product:hypothetical protein	PI:*AGC35182.1*<br>Product:hypothetical protein
PI:*QXN76341.1*<br>Product:hypothetical protein	PI:*AGC35181.1*<br>Product:hypothetical protein

PI:*QXN76343.1*<br>Product:terminase large subunit	PI:*AGC35180.1*<br>Product:large terminase protein
PI:*QXN76344.1*<br>Product:hypothetical protein	PI:*AGC35179.1*<br>Product:hypothetical protein
PI:*QXN76345.1*<br>Product:hypothetical protein	PI:*AGC35178.1*<br>Product:hypothetical protein
PI:*QXN76346.1*<br>Product:recombination and repair protein	PI:*AGC35177.1*<br>Product:recombination and repair protein
PI:*QXN76347.1*<br>Product:hypothetical protein	PI:*AGC35176.1*<br>Product:hypothetical protein
PI:*QXN76348.1*<br>Product:exodeoxyribonuclease X	PI:*AGC35175.1*<br>Product:putative exonuclease
PI:*QXN76349.1*<br>Product:ATP-dependent DNA helicase	PI:*AGC35174.1*<br>Product:helicase
PI:*QXN76350.1*<br>Product:hypothetical protein	PI:*AGC35173.1*<br>Product:hypothetical protein
PI:*QXN76351.1*<br>Product:hypothetical protein	PI:*AGC35172.1*<br>Product:hypothetical protein
PI:*QXN76352.1*<br>Product:hypothetical protein	PI:*AGC35171.1*<br>Product:hypothetical protein
PI:*QXN76353.1*<br>Product:hypothetical protein	PI:*AGC35170.1*<br>Product:hypothetical protein
PI:*QXN76354.1*<br>Product:hypothetical protein	PI:*AGC35169.1*<br>Product:hypothetical protein
PI:*QXN76355.1*<br>Product:hypothetical protein	PI:*AGC35168.1*<br>Product:hypothetical protein
PI:*QXN76356.1*<br>Product:hypothetical protein	PI:*AGC35167.1*<br>Product:hypothetical protein
PI:*QXN76357.1*<br>Product:hypothetical protein	PI:*AGC35166.1*<br>Product:hypothetical protein
PI:*QXN76358.1*<br>Product:DNA primase subunit	PI:*AGC35165.1*<br>Product:DNA primase subunit
PI:*QXN76359.1*<br>Product:helicase	PI:*AGC35164.1*<br>Product:DNA primase-helicase subunit
PI:*QXN76360.1*<br>Product:hypothetical protein	PI:*AGC35163.1*<br>Product:hypothetical protein
PI:*QXN76361.1*<br>Product:hypothetical protein	PI:*AGC35162.1*<br>Product:hypothetical protein
PI:*QXN76362.1*<br>Product:hypothetical protein	PI:*AGC35161.1*<br>Product:hypothetical protein
PI:*QXN76363.1*<br>Product:hypothetical protein	PI:*AGC35160.1*<br>Product:hypothetical protein
PI:*QXN76364.1*<br>Product:hypothetical protein	PI:*AGC35159.1*<br>Product:hypothetical protein
PI:*QXN76365.1*<br>Product:hypothetical protein	PI:*AGC35158.1*<br>Product:hypothetical protein
PI:*QXN76366.1*<br>Product:hypothetical protein	PI:*AGC35157.1*<br>Product:hypothetical protein

PI:*QXN76367.1* Product:hypothetical protein	PI:*AGC35156.1* Product:hypothetical protein
PI:*QXN76368.1* Product:exonuclease	PI:*AGC35155.1* Product:putative exonuclease
PI:*QXN76369.1* Product:hypothetical protein	PI: *GC35154.1* Product:hypothetical protein
PI:*QXN76370.1* Product:hypothetical protein	PI:*AGC35153.1* Product:hypothetical protein
PI:*QXN76371.1* Product:aerobic ribonucleoside-diphosphate reductase	PI:*AGC35152.1* Product:aerobic ribonucleoside diphosphate reductase
PI:*QXN76373.1* Product:aerobic ribonucleoside-diphosphate reductase	PI:*AGC35151.1* Product:aerobic ribonucleoside diphosphate reductase
PI:*QXN76374.1* Product:hypothetical protein	PI:*AGC35150.1* Product:hypothetical protein
PI:*QXN76375.1* Product:hypothetical protein	PI:*AGC35149.1* Product:hypothetical protein
PI:*QXN76376.1* Product:hypothetical protein	PI:*AGC35148.1* Product:hypothetical protein
PI:*QXN76377.1* Product:hypothetical protein	PI:*AGC35147.1* Product:hypothetical protein
PI:*QXN76378.1* Product:hypothetical protein	PI:*AGC35146.1* Product:hypothetical protein
PI:*QXN76380.1* Product:hypothetical protein	PI:*AGC35145.1* Product:hypothetical protein
PI:*QXN76381.1* Product:hypothetical protein	PI:*AGC35144.1* Product:hypothetical protein
PI:*QXN76382.1* Product:dihydrofolate reductase	PI:*AGC35143.1* Product:dihydrofolate reductase
PI:*QXN76383.1* Product:ribonuclease H	PI:*AGC35142.1* Product:ribonuclease HI
PI:*QXN76384.1* Product:ATP-dependent DNA helicase	PI:*AGC35141.1* Product:DNA helicase
PI:*QXN76385.1* Product:hypothetical protein	PI:*AGC35140.1* Product:hypothetical protein
PI:*QXN76386.1* Product:hypothetical protein	PI:*AGC35139.1* Product:hypothetical protein
PI:*QXN76387.1* Product:hypothetical protein	PI:*AGC35138.1* Product:hypothetical protein
PI:*QXN76388.1* Product:hypothetical protein	PI:*AGC35137.1* Product:hypothetical protein
PI:*QXN76389.1* Product:hypothetical protein	PI:*AGC35136.1* Product:hypothetical protein
PI:*QXN76390.1* Product:hypothetical protein	PI:*AGC35135.1* Product:hypothetical protein
PI:*QXN76391.1* Product:hypothetical protein	PI:*AGC35134.1* Product:hypothetical protein

PI:*QXN76392.1*<br>Product:topoisomerase II large subunit	PI:*AGC35133.1*<br>Product:topoisomerase II large subunit
PI:*QXN76393.1*<br>Product:topoisomerase II medium subunit	PI:*AGC35132.1*<br>Product:topoisomerase II medium subunit
PI:*QXN76394.1*<br>Product:hypothetical protein	PI:*AGC35131.1*<br>Product:hypothetical protein
PI:*QXN76395.1*<br>Product:hypothetical protein	PI:*AGC35130.1*<br>Product:hypothetical protein
PI:*QXN76396.1*<br>Product:hypothetical protein	PI:*AGC35129.1*<br>Product:hypothetical protein
PI:*QXN76397.1*<br>Product:hypothetical protein	PI:*AGC35128.1*<br>Product:hypothetical protein
PI:*QXN76398.1*<br>Product:hypothetical protein	PI:*AGC35127.1*<br>Product:hypothetical protein
PI:*QXN76399.1*<br>Product:hypothetical protein	PI:*AGC35126.1*<br>Product:hypothetical protein
PI:*QXN76400.1*<br>Product:replication factor C small subunit	PI:*AGC35125.1*<br>Product:clamp loader subunit DNA polymerase accessory
PI:*QXN76401.1*<br>Product:hypothetical protein	PI:*AGC35124.1*<br>Product:hypothetical protein
PI:*QXN76402.1*<br>Product:hypothetical protein	PI:*AGC35123.1*<br>Product:hypothetical protein
PI:*QXN76403.1*<br>Product:hypothetical protein	PI:*AGC35122.1*<br>Product:hypothetical protein
PI:*QXN76404.1*<br>Product:hypothetical protein	PI:*AGC35121.1*<br>Product:hypothetical protein
PI:*QXN76405.1*<br>Product:hypothetical protein	PI:*AGC35120.1*<br>Product:hypothetical protein
PI:*QXN76406.1*<br>Product:hypothetical protein	PI:*AGC35119.1*<br>Product:hypothetical protein
PI:*QXN76407.1*<br>Product:peptidase	PI:*AGC35118.1*<br>Product:putative peptidase
PI:*QXN76408.1*<br>Product:hypothetical protein	PI:*AGC35117.1*<br>Product:hypothetical protein
PI:*QXN76409.1*<br>Product:hypothetical protein	PI:*AGC35116.1*<br>Product:hypothetical protein
PI:*QXN76410.1*<br>Product:hypothetical protein	PI:*AGC35115.1*<br>Product:hypothetical protein
PI:*QXN76411.1*<br>Product:hypothetical protein	PI:*AGC35114.1*<br>Product:hypothetical protein
PI:*QXN76412.1*<br>Product:hypothetical protein	PI:*AGC35113.1*<br>Product:hypothetical protein
PI:*QXN76413.1*<br>Product:hypothetical protein	PI:*AGC35112.1*<br>Product:hypothetical protein
PI:*QXN76414.1*<br>Product:hypothetical protein	PI:*AGC35111.1*<br>Product:hypothetical protein

PI:*QXN76415.1*<br>Product:hypothetical protein	PI:*AGC35110.1*<br>Product:hypothetical protein
PI:*QXN76417.1*<br>Product:hypothetical protein	PI:*AGC35109.1*<br>Product:hypothetical protein
PI:*QXN76418.1*<br>Product:hypothetical protein	PI:*AGC35108.1*<br>Product:hypothetical protein
PI:*QXN76419.1*<br>Product:hypothetical protein	PI:*AGC35107.1*<br>Product:hypothetical protein
PI:*QXN76420.1*<br>Product:hypothetical protein	PI:*AGC35106.1*<br>Product:hypothetical protein
PI:*QXN76421.1*<br>Product:hypothetical protein	PI:*AGC35105.1*<br>Product:hypothetical protein
PI:*QXN76422.1*<br>Product:hypothetical protein	PI:*AGC35104.1*<br>Product:hypothetical protein
PI:*QXN76423.1*<br>Product:hypothetical protein	PI:*AGC35103.1*<br>Product:hypothetical protein
PI:*QXN76424.1*<br>Product:hypothetical protein	PI:*AGC35102.1*<br>Product:hypothetical protein
PI:*QXN76425.1*<br>Product:hypothetical protein	PI:*AGC35101.1*<br>Product:hypothetical protein
PI:*QXN76426.1*<br>Product:hypothetical protein	PI:*AGC35100.1*<br>Product:hypothetical protein
PI:*QXN76427.1*<br>Product:hypothetical protein	PI:*AGC35099.1*<br>Product:hypothetical protein
PI:*QXN76428.1*<br>Product:hypothetical protein	PI:*AGC35098.1*<br>Product:hypothetical protein
PI:*QXN76430.1*<br>Product:hypothetical protein	PI:*AGC35097.1*<br>Product:hypothetical protein
PI:*QXN76431.1*<br>Product:hypothetical protein	PI:*AGC35096.1*<br>Product:putative DNA-methyltransferase
PI:*QXN76432.1*<br>Product:hypothetical protein	PI:*AGC35095.1*<br>Product:hypothetical protein
PI:*QXN76433.1*<br>Product:hypothetical protein	PI:*AGC35094.1*<br>Product:hypothetical protein
PI:*QXN76434.1*<br>Product:hypothetical protein	PI:*AGC35093.1*<br>Product:hypothetical protein
PI:*QXN76435.1*<br>Product:hypothetical protein	PI:*AGC35092.1*<br>Product:hypothetical protein
PI:*QXN76436.1*<br>Product:hypothetical protein	PI:*AGC35091.1*<br>Product:hypothetical protein
PI:*QXN76437.1*<br>Product:hypothetical protein	PI:*AGC35090.1*<br>Product:hypothetical protein
PI:*QXN76438.1*<br>Product:hypothetical protein	PI:*AGC35089.1*<br>Product:hypothetical protein
PI:*QXN76439.1*<br>Product:hypothetical protein	PI:*AGC35088.1*<br>Product:hypothetical protein
PI:*QXN76440.1*<br>Product:hypothetical protein	PI:*AGC35087.1*<br>Product:hypothetical protein

PI:*QXN76441.1*<br>Product:hypothetical protein	PI:*AGC35086.1*<br>Product:hypothetical protein
PI:*QXN76442.1*<br>Product:hypothetical protein	PI:*AGC35085.1*<br>Product:hypothetical protein
PI:*QXN76443.1*<br>Product:hypothetical protein	PI:*AGC35084.1*<br>Product:hypothetical protein
PI:*QXN76444.1*<br>Product:hypothetical protein	PI:*AGC35083.1*<br>Product:hypothetical protein
PI:*QXN76445.1*<br>Product:hypothetical protein	PI:*AGC35082.1*<br>Product:hypothetical protein
PI:*QXN76446.1*<br>Product:hypothetical protein	PI:*AGC35081.1*<br>Product:hypothetical protein
PI:*QXN76447.1*<br>Product:hypothetical protein	PI:*AGC35080.1*<br>Product:hypothetical protein
PI:*QXN76448.1*<br>Product:hypothetical protein	PI:*AGC35079.1*<br>Product:hypothetical protein
PI:*QXN76449.1*<br>Product:hypothetical protein	PI:*AGC35078.1*<br>Product:hypothetical protein
PI:*QXN76450.1*<br>Product:hypothetical protein	PI:*AGC35077.1*<br>Product:hypothetical protein
PI:*QXN76451.1*<br>Product:hypothetical protein	PI:*AGC35076.1*<br>Product:hypothetical protein
PI:*QXN76452.1*<br>Product:hypothetical protein	PI:*AGC35075.1*<br>Product:hypothetical protein
PI:*QXN76453.1*<br>Product:hypothetical protein	PI:*AGC35074.1*<br>Product:hypothetical protein
PI:*QXN76454.1*<br>Product:hypothetical protein	PI:*AGC35073.1*<br>Product:hypothetical protein
PI:*QXN76455.1*<br>Product:hypothetical protein	PI:*AGC35071.1*<br>Product:hypothetical protein
PI:*QXN76456.1*<br>Product:hypothetical protein	PI:*AGC35070.1*<br>Product:hypothetical protein
PI:*QXN76458.1*<br>Product:hypothetical protein	PI:*AGC35069.1*<br>Product:hypothetical protein
PI:*QXN76459.1*<br>Product:hypothetical protein	PI:*AGC35068.1*<br>Product:hypothetical protein
PI:*QXN76460.1*<br>Product:hypothetical protein	PI:*AGC35067.1*<br>Product:hypothetical protein
PI:*QXN76462.1*<br>Product:hypothetical protein	PI:*AGC35066.1*<br>Product:hypothetical protein
PI:*QXN76463.1*<br>Product:hypothetical protein	PI:*AGC35065.1*<br>Product:hypothetical protein
PI:*QXN76465.1*<br>Product:hypothetical protein	PI:*AGC35064.1*<br>Product:hypothetical protein
PI:*QXN76466.1*<br>Product:hypothetical protein	PI:*AGC35063.1*<br>Product:hypothetical protein
PI:*QXN76467.1*<br>Product:hypothetical protein	PI:*AGC35062.1*<br>Product:hypothetical protein

PI:*QXN76468.1* Product:hypothetical protein	PI:*AGC35061.1* Product:hypothetical protein
PI:*QXN76469.1* Product:hypothetical protein	PI:*AGC35060.1* Product:hypothetical protein
PI:*QXN76470.1* Product:hypothetical protein	PI:*AGC35059.1* Product:hypothetical protein
PI:*QXN76471.1* Product:hypothetical protein	PI:*AGC35058.1* Product:hypothetical protein
PI:*QXN76472.1* Product:hypothetical protein	PI:*AGC35057.1* Product:hypothetical protein
PI:*QXN76473.1* Product:hypothetical protein	PI:*AGC35056.1* Product:hypothetical protein
PI:*QXN76474.1* Product:hypothetical protein	PI:*AGC35055.1* Product:hypothetical protein
PI:*QXN76475.1* Product:hypothetical protein	PI:*AGC35054.1* Product:hypothetical protein
PI:*QXN76476.1* Product:hypothetical protein	PI:*AGC35053.1* Product:hypothetical protein
PI:*QXN76477.1* Product:hypothetical protein	PI:*AGC35052.1* Product:hypothetical protein
PI:*QXN76478.1* Product:hypothetical protein	PI:*AGC35051.1* Product:hypothetical protein
PI:*QXN76479.1* Product:hypothetical protein	PI:*AGC35050.1* Product:hypothetical protein
PI:*QXN76480.1* Product:hypothetical protein	PI:*AGC35049.1* Product:hypothetical protein
PI:*QXN76481.1* Product:hypothetical protein	PI:*AGC35048.1* Product:hypothetical protein
PI:*QXN76482.1* Product:hypothetical protein	PI:*AGC35047.1* Product:hypothetical protein
PI:*QXN76483.1* Product:hypothetical protein	PI:*AGC35046.1* Product:hypothetical protein
PI:*QXN76484.1* Product:hypothetical protein	PI:*AGC35045.1* Product:hypothetical protein
PI:*QXN76485.1* Product:hypothetical protein	PI:*AGC35044.1* Product:hypothetical protein
PI:*QXN76486.1* Product:hypothetical protein	PI:*AGC35043.1* Product:hypothetical protein
PI:*QXN76487.1* Product:hypothetical protein	PI:*AGC35042.1* Product:hypothetical protein
PI:*QXN76488.1* Product:hypothetical protein	PI:*AGC35041.1* Product:hypothetical protein
PI:*QXN76489.1* Product:hypothetical protein	PI:*AGC35040.1* Product:hypothetical protein
PI:*QXN76490.1* Product:hypothetical protein	PI:*AGC35039.1* Product:hypothetical protein
PI:*QXN76491.1* Product:hypothetical protein	PI:*AGC35038.1* Product:hypothetical protein

PI:*QXN76492.1*<br>Product:hypothetical protein	PI:*AGC35037.1*<br>Product:hypothetical protein
PI:*QXN76493.1*<br>Product:RNA polymerase sigma factor	PI:*AGC35036.1*<br>Product:putative RNA polymerase sigma factor
PI:*QXN76494.1*<br>Product:hypothetical protein	PI:*AGC35035.1*<br>Product:hypothetical protein
PI:*QXN76495.1*<br>Product:hypothetical protein	PI:*AGC35034.1*<br>Product:hypothetical protein
PI:*QXN76496.1*<br>Product:hypothetical protein	PI:*AGC35033.1*<br>Product:hypothetical protein
PI:*QXN76497.1*<br>Product:hypothetical protein	PI:*AGC35032.1*<br>Product:hypothetical protein
PI:*QXN76498.1*<br>Product:hypothetical protein	PI:*AGC35031.1*<br>Product:hypothetical protein
PI:*QXN76499.1*<br>Product:hypothetical protein	PI:*AGC35030.1*<br>Product:hypothetical protein
PI:*QXN76500.1*<br>Product:hypothetical protein	PI:*AGC35029.1*<br>Product:hypothetical protein
PI:*QXN76501.1*<br>Product:hypothetical protein	PI:*AGC35028.1*<br>Product:hypothetical protein
PI:*QXN76502.1*<br>Product:hypothetical protein	PI:*AGC35027.1*<br>Product:hypothetical protein
PI:*QXN76503.1*<br>Product:hypothetical protein	PI:*AGC35026.1*<br>Product:hypothetical protein
PI:*QXN76504.1*<br>Product:hypothetical protein	PI:*AGC35025.1*<br>Product:hypothetical protein
PI:*QXN76505.1*<br>Product:hypothetical protein	PI:*AGC35024.1*<br>Product:hypothetical protein
PI:*QXN76506.1*<br>Product:hypothetical protein	PI:*AGC35023.1*<br>Product:hypothetical protein
PI:*QXN76507.1*<br>Product:hypothetical protein	PI:*AGC35022.1*<br>Product:hypothetical protein
PI:*QXN76508.1*<br>Product:hypothetical protein	PI:*AGC35021.1*<br>Product:hypothetical protein
PI:*QXN76509.1*<br>Product:hypothetical protein	PI:*AGC35020.1*<br>Product:hypothetical protein
PI:*QXN76510.1*<br>Product:hypothetical protein	PI:*AGC35019.1*<br>Product:hypothetical protein
PI:*QXN76511.1*<br>Product:hypothetical protein	PI:*AGC35018.1*<br>Product:hypothetical protein
PI:*QXN76513.1*<br>Product:hypothetical protein	PI:*AGC35017.1*<br>Product:hypothetical protein
PI:*QXN76514.1*<br>Product:hypothetical protein	PI:*AGC35016.1*<br>Product:hypothetical protein
PI:*QXN76515.1*<br>Product:hypothetical protein	PI:*AGC35015.1*<br>Product:hypothetical protein
PI:*QXN76516.1*<br>Product:hypothetical protein	PI:*AGC35014.1*<br>Product:hypothetical protein

PI:*QXN76517.1*<br>Product:hypothetical protein	PI:*AGC35013.1*<br>Product:hypothetical protein
PI:*QXN76518.1*<br>Product:hypothetical protein	PI:*AGC35012.1*<br>Product:hypothetical protein
PI:*QXN76519.1*<br>Product:hypothetical protein	PI:*AGC35011.1*<br>Product:hypothetical protein
PI:*QXN76520.1*<br>Product:hypothetical protein	PI:*AGC35010.1*<br>Product:hypothetical protein
PI:*QXN76521.1*<br>Product:hypothetical protein	PI:*AGC35009.1*<br>Product:hypothetical protein
PI:*QXN76522.1*<br>Product:hypothetical protein	PI:*AGC35008.1*<br>Product:hypothetical protein
PI:*QXN76524.1*<br>Product:hypothetical protein	PI:*AGC35007.1*<br>Product:hypothetical protein
PI:*QXN76525.1*<br>Product:hypothetical protein	PI:*AGC35006.1*<br>Product:hypothetical protein
PI:*QXN76526.1*<br>Product:hypothetical protein	PI:*AGC35005.1*<br>Product:hypothetical protein
PI:*QXN76527.1*<br>Product:hypothetical protein	PI:*AGC35004.1*<br>Product:hypothetical protein
PI:*QXN76530.1*<br>Product:hypothetical protein	PI:*AGC35003.1*<br>Product:hypothetical protein
PI:*QXN76531.1*<br>Product:hypothetical protein	PI:*AGC35002.1*<br>Product:hypothetical protein
PI:*QXN76532.1*<br>Product:hypothetical protein	PI:*AGC35001.1*<br>Product:hypothetical protein
PI:*QXN76533.1*<br>Product:hypothetical protein	PI:*AGC35000.1*<br>Product:hypothetical protein
PI:*QXN76534.1*<br>Product:hypothetical protein	PI:*AGC34998.1*<br>Product:hypothetical protein
PI:*QXN76535.1*<br>Product:hypothetical protein	PI:*AGC34997.1*<br>Product:hypothetical protein
PI:*QXN76536.1*<br>Product:hypothetical protein	PI:*AGC34996.1*<br>Product:hypothetical protein
PI:*QXN76538.1*<br>Product:hypothetical protein	PI:*AGC34995.1*<br>Product:hypothetical protein
PI:*QXN76539.1*<br>Product:hypothetical protein	PI:*AGC34994.1*<br>Product:hypothetical protein
PI:*QXN76541.1*<br>Product:hypothetical protein	PI:*AGC34993.1*<br>Product:hypothetical protein
PI:*QXN76542.1*<br>Product:hypothetical protein	PI:*AGC34992.1*<br>Product:hypothetical protein
PI:*QXN76544.1*<br>Product:hypothetical protein	PI:*AGC34991.1*<br>Product:hypothetical protein
PI:*QXN76545.1*<br>Product:hypothetical protein	PI:*AGC34990.1*<br>Product:hypothetical protein

PI:*QXN76547.1* Product:endonuclease	PI:*AGC34989.1* Product:endonuclease V N-glycosylase UV repair enzyme
PI:*QXN76548.1* Product:hypothetical protein	PI:*AGC34987.1* Product:hypothetical protein
PI:*QXN76551.1* Product:hypothetical protein	PI:*AGC34984.1* Product:hypothetical protein
PI:*QXN76552.1* Product:hypothetical protein	PI:*AGC34983.1* Product:hypothetical protein
PI:*QXN76553.1* Product:hypothetical protein	PI:*AGC34982.1* Product:hypothetical protein
PI:*QXN76554.1* Product:hypothetical protein	PI:*AGC34981.1* Product:hypothetical protein
PI:*QXN76555.1* Product:hypothetical protein	PI:*AGC34980.1* Product:hypothetical protein
PI:*QXN76556.1* Product:hypothetical protein	PI:*AGC34979.1* Product:hypothetical protein
PI:*QXN76558.1* Product:hypothetical protein	PI:*AGC34978.1* Product:hypothetical protein
PI:*QXN76560.1* Product:hypothetical protein	PI:*AGC34977.1* Product:hypothetical protein
PI:*QXN76563.1* Product:hypothetical protein	PI:*AGC34968.1* Product:hypothetical protein
PI:*QXN76567.1* Product:hypothetical protein	PI:*AGC34970.1* Product:hypothetical protein
PI:*QXN76568.1* Product:regulator of chromosome condensation-like	PI:*AGC34972.1* Product:regulator of chromosome condensation-like
PI:*QXN76569.1* Product:hypothetical protein	PI:*AGC34967.1* Product:hypothetical protein
PI:*QXN76572.1* Product:hypothetical protein	PI:*AGC34966.1* Product:regulator of chromosome condensation RCC1
PI:*QXN76574.1* Product:regulator of chromosome condensation	PI:*AGC34969.1* Product:regulator of chromosome condensation RCC1
PI:*QXN76580.1* Product:hypothetical protein	PI:*AGC34964.1* Product:hypothetical protein
PI:*QXN76583.1* Product:hypothetical protein	PI:*AGC34962.1* Product:hypothetical protein
PI:*QXN76584.1* Product:hypothetical protein	PI:*AGC34961.1* Product:hypothetical protein
PI:*QXN76587.1* Product:hypothetical protein	PI:*AGC34960.1* Product:hypothetical protein
PI:*QXN76588.1* Product:hypothetical protein	PI:*AGC34959.1* Product:hypothetical protein
PI:*QXN76589.1* Product:hypothetical protein	PI:*AGC34958.1* Product:hypothetical protein
PI:*QXN76590.1* Product:hypothetical protein	PI:*AGC34957.1* Product:hypothetical protein

PI:*QXN76591.1* Product:hypothetical protein	PI:*AGC34956.1* Product:hypothetical protein
PI:*QXN76592.1* Product:hypothetical protein	PI:*AGC34955.1* Product:hypothetical protein
PI:*QXN76593.1* Product:hypothetical protein	PI:*AGC34954.1* Product:hypothetical protein
PI:*QXN76594.1* Product:hypothetical protein	PI:*AGC34953.1* Product:hypothetical protein
PI:*QXN76595.1* Product:hypothetical protein	PI:*AGC34952.1* Product:hypothetical protein
PI:*QXN76596.1* Product:hypothetical protein	PI:*AGC34949.1* Product:hypothetical protein
PI:*QXN76598.1* Product:hypothetical protein	PI:*AGC34948.1* Product:hypothetical protein
PI:*QXN76600.1* Product:hypothetical protein	PI:*AGC34947.1* Product:hypothetical protein
PI:*QXN76601.1* Product:hypothetical protein	PI:*AGC34946.1* Product:hypothetical protein
PI:*QXN76603.1* Product:hypothetical protein	PI:*AGC34945.1* Product:hypothetical protein
PI:*QXN76604.1* Product:hypothetical protein	PI:*AGC34944.1* Product:hypothetical protein
PI:*QXN76605.1* Product:hypothetical protein	PI:*AGC34943.1* Product:hypothetical protein
PI:*QXN76606.1* Product:hypothetical protein	PI:*AGC34942.1* Product:hypothetical protein
PI:*QXN76607.1* Product:hypothetical protein	PI:*AGC34941.1* Product:hypothetical protein
PI:*QXN76609.1* Product:hypothetical protein	PI:*AGC34939.1* Product:hypothetical protein
PI:*QXN76610.1* Product:hypothetical protein	PI:*AGC34938.1* Product:hypothetical protein
PI:*QXN76611.1* Product:hypothetical protein	PI:*AGC34937.1* Product:hypothetical protein
PI:*QXN76613.1* Product:hypothetical protein	PI:*AGC34936.1* Product:hypothetical protein
PI:*QXN76614.1* Product:hypothetical protein	PI:*AGC34935.1* Product:hypothetical protein
PI:*QXN76615.1* Product:hypothetical protein	PI:*AGC34934.1* Product:hypothetical protein
PI:*QXN76616.1* Product:hypothetical protein	PI:*AGC34933.1* Product:hypothetical protein
PI:*QXN76618.1* Product:hypothetical protein	PI:*AGC34932.1* Product:hypothetical protein
PI:*QXN76619.1* Product:hypothetical protein	PI:*AGC34931.1* Product:hypothetical protein
PI:*QXN76620.1* Product:hypothetical protein	PI:*AGC34930.1* Product:hypothetical protein

PI:*QXN76622.1*<br>Product:hypothetical protein	PI:*AGC34929.1*<br>Product:hypothetical protein
PI:*QXN76623.1*<br>Product:hypothetical protein	PI:*AGC34928.1*<br>Product:hypothetical protein
PI:*QXN76624.1*<br>Product:hypothetical protein	PI:*AGC34926.1*<br>Product:hypothetical protein
PI:*QXN76625.1*<br>Product:hypothetical protein	PI:*AGC34925.1*<br>Product:hypothetical protein
PI:*QXN76626.1*<br>Product:hypothetical protein	PI:*AGC34924.1*<br>Product:hypothetical protein
PI:*QXN76627.1*<br>Product:hypothetical protein	PI:*AGC34923.1*<br>Product:hypothetical protein
PI:*QXN76630.1*<br>Product:hypothetical protein	PI:*AGC34922.1*<br>Product:hypothetical protein
PI:*QXN76631.1*<br>Product:hypothetical protein	PI:*AGC34921.1*<br>Product:hypothetical protein
PI:*QXN76632.1*<br>Product:hypothetical protein	PI:*AGC34920.1*<br>Product:hypothetical protein
PI:*QXN76633.1*<br>Product:hypothetical protein	PI:*AGC34919.1*<br>Product:hypothetical protein
PI:*QXN76634.1*<br>Product:hypothetical protein	PI:*AGC34918.1*<br>Product:hypothetical protein
PI:*QXN76635.1*<br>Product:hypothetical protein	PI:*AGC34917.1*<br>Poduct:hypothetical protein
PI:*QXN76636.1*<br>Product:hypothetical protein	PI:*AGC34916.1*<br>Product:hypothetical protein
PI:*QXN76638.1*<br>Product:hypothetical protein	PI:*AGC34915.1*<br>Product:hypothetical protein
PI:*QXN76639.1*<br>Product:hypothetical protein	PI:*AGC34914.1*<br>Product:hypothetical protein
PI:*QXN76640.1*<br>Product:hypothetical protein	PI:*AGC34913.1*<br>Product:hypothetical protein
PI:*QXN76641.1*<br>Product:hypothetical protein	PI:*AGC34912.1*<br>Product:hypothetical protein
